# Supplementary material for: Structures of Saccharolobus solfataricus initiation complexes with leaderless mRNAs highlight archaeal features and eukaryotic proximity
Source: Nat Commun. 2025 Jan 2;16:348. doi: 10.1038/s41467-024-55718-5 (PMC11698992; doi:10.1038/s41467-024-55718-5)
Supplement: Supplementary file 1 — Supplementary Information [file 41467_2024_55718_MOESM1_ESM.pdf]

Supplementary Information

Structures of *Saccharolobus solfataricus* Initiation Complexes with Leaderless mRNAs Highlight Archaeal Features and Eukaryotic Proximity

Gabrielle Bourgeois*, Pierre-Damien Coureux*, Christine Lazennec-Schurdevin, Clément Madru, Thomas Gaillard, Magalie Duchateau, Julia Chamot-Rooke, Sophie Bourcier, Yves Mechulam and Emmanuelle Schmitt

Supplementary Methods

Supplementary Tables

Supplementary Figures

Supplementary References

Supplementary Methods

Grid inhomogeneous solvation theory analysis of 16S rRNA G889

Water density around G889 nucleobase of 16S rRNA was studied by explicit water molecular dynamics (MD) simulation, followed by grid inhomogeneous solvation theory (GIST)[1, 2] analysis. GIST is a method giving access to the structure and thermodynamics of solvent in the vicinity of a solute molecule. The ribosomal molecular system was truncated at 12 Å around G889 nucleobase, resulting in 36 RNA nucleotides, 36 experimentally placed water oxygens, and 3 magnesium ions (Supplementary Table 6). Two alternative conformations were considered for the 5'-terminal triphosphate nucleotide (A3P) of mRNA, close in space to G889. Hydrogen atoms were added and their positions optimized with the Reduce program[3]. The system was immersed in a truncated octahedron box extending 10 Å away from the solute and filled with water molecules. Supplementary Figure 16A shows the simulated system. Minimization was conducted, starting with the hydrogen atoms alone, then adding the solvent, while gradually reducing the harmonic positional restraints on the rest, until reaching a force constant of 10 kcal/mol/Å^2. The reference for positional restraints was the initial conformation. Only heavy atoms of the solute and magnesium ions were restrained. Minimizations were stopped when the RMSD of the gradient components was less than 0.1 kcal/mol/Å. MD simulation was then conducted. Heating to 300 K was first performed, in the constant volume and temperature (NVT) ensemble. Equilibration was then performed in the constant pressure and temperature (NPT) ensemble during 2 ns, then in the NVT ensemble during 10 ns. The production simulation was then run in the NVT ensemble during 100 ns and conformations were saved every 4 ps. The harmonic positional restraints used in final minimization were maintained during MD simulation. The integration time step was 2 fs. Bonds involving hydrogen atoms were constrained with the SHAKE algorithm[4]. Periodic boundary conditions were applied. The Particle Mesh Ewald (PME) method[5] was used to handle long-range electrostatic interactions, while long-range van der Waals interactions were estimated by a continuum model. A cutoff distance of 9 Å was used for direct calculation of interactions. Temperature was controlled with a Langevin thermostat[6], targeting a temperature of 300 K and using a collision frequency of 2 ps^{-1}. Pressure was controlled with the Berendsen barostat[7], targeting a pressure of 1 bar and using a relaxation time of 2 ps. The AMBER

suite[8] of programs (version 21) was employed, with the SANDER program for minimization and heating, and the GPU accelerated version of the PMEMD program[9, 10] for equilibration and production. The ff99OL3 force field[11, 12] was used for RNA, along with modrna08[13] for modified nucleotides, Carlson ATP parameters[14] were adapted for the A3P nucleotide, TIP3P[15] was used for water, and Li and Merz parameters[16] (12-6 normal usage set) for magnesium ions. For each simulation condition, three replicates were run, differing in their random initial velocities. The production simulation trajectories were then analyzed with GIST around G889 nucleobase, as implemented in the CPPTRAJ program[17]. The grid center was defined as the origin of the reference Cartesian frame of G889 nucleobase, following Kalra et al.g[18]. The grid dimensions were 30, 30, and 36, in the x, y, and z directions (MD simulation axes), while the grid spacing was 0.5 Å. Reference density for water was 0.0329 molecules/Å^3. Supplementary Figure 16B shows the simulated system without water molecules, along with water oxygen and hydrogen densities obtained by GIST. Supplementary Figure 16C is a zoomed view on a region above G889 nucleobase. This region is defined along the x', y', z' axes of the reference Cartesian frame[18] of G889 nucleobase, as x'=[-2.5,4.5], y'=[-2.5,2.5], z'=[-4.5,0] Å (gray edges in Supplementary Figure 16C). Water oxygen and hydrogen densities are shown to be important (more than 2 times the bulk water density) in the z'=[-4,-2.5] Å region (black edges in Supplementary Figure 16C). This region contains five experimentally placed water molecules (red spheres in Supplementary Figure 16C and Figure 7D). Supplementary Table 7 presents a selection of water properties calculated with GIST, averaged over these different regions. Properties are water oxygen and hydrogen densities, as well as solute-water and water-water interaction energy densities. These quantitative results show that when gradually focusing on the region above G889 nucleobase, water density increases, as well as both solute-water and water-water interaction energy densities. At the location of the five water molecules that have been placed based on the experimental electronic density, water oxygen and hydrogen densities are 3.7 and 2.0 times higher than bulk water density, while solute-water and water-water interaction energy densities are -1.6 and -0.6 kcal/mol/Å^3. Robustness and convergence of the GIST analysis was assessed. Redoing the MD simulations without the 36 experimentally placed water oxygens (Supplementary Table 7) leads to almost identical results to those obtained including experimental water oxygens, indicating that initial placement of water

molecules has limited influence. Redoing the GIST analysis on 25% shorter MD simulations (Supplementary Table 8) leads to almost identical results, indicating convergence with respect to simulation length. At last, simulation of each condition was triplicated with different random initial velocities, showing very limited dispersion of results (standard deviations in Supplementary Tables 7 to 9). These analyses suggest that MD simulations have sufficiently sampled the water conformational space. Overall, these results support the assignment of the five water molecules in the region above G889 nucleobase. Favorable interaction energies of these water molecules with G889 is calculated. These water positions are compatible with OH and/or lone pair stacking interactions with G889, as studied by Kalra et al.[18] with quantum chemical calculations. At last, we note that the shape of water density above G889 nucleobase is suggestive of a purine nucleobase, where the cycle atoms would be replaced by a network of water molecules.

Supplementary Tables

mRNAs	Size	Sequences 5'>3'	Production-purification
model-SD	658	GGGAGACCACAACGGUUUCCCTCT<br>AGAAAGUAGGGUUAGGCAGAAAUU<br>UGGAGGUGAUUUAAAUGCCAAAGG<br>AGAAGCCCCACGUUAACAUCGUGU<br>UUAUCGGACACGUAGACCACGGAA<br>AGAGCACGACCAUCGGAAGGCUCC<br>UCUACGACACCGGGAACAUCCCAG<br>AGACCAUCAUCAAGAAGUUCGA…	[19]
Ss-aEF1A-like	182	GUUAAAGUGAUUUGAUGUAAUGA<br>AAUUACCCCGAGAGAAUGAUGAAG<br>GAAAAGUAGAAUAUAAGCUUAUUC<br>UUUCAAGUGUAACCCCGGAUCGUC<br>UUCAAGAAUUAGCUACUCAAAUGA<br>AGUAUAGGUUAGAGGAAGGUGAU<br>GGUGAAGCAUUUUACGUUAUAGGU<br>GUAAGUGAUGAAGGCG	Cloned into pET3a BglII-XhoI<br>In vitro transcription<br>MonoQ
Ss-MAP	161	5'-OH-<br>AUGACUGAGGAUGAACUCAAUAAG<br>CUUUUAUUAGCAGGUAAGAUUGCA<br>GCUAAGGCUAGAGAUGAAGUUUCA<br>UUAGACGUUAAAGCUAGUGCUAAG<br>GUUUUAGAUAUUUGUGAAGAGGU<br>UGAAAGUAUAAUAAUCGAAAAUAA<br>AGCGUUUCCAUCAUUUCC	Cloned into pET3a XbaI-XhoI<br>5' hammerhead ribozyme<br>In vitro transcription<br>MonoQ
Ss-aIF2β	171	5'PPP-<br>GUGAGUUCAGAAAAAGAAUACGUA<br>GAAAUGCUUGAUAGGCUAUACUCG<br>AAAUUACCAGAAAAAGGACGUAAG<br>GAAGGUACACAAUCAUUGCCUAAU<br>AUGAUAAUACUCAAUAUAGGAAAU<br>ACUACUAUAAUUAGAAACUUUGCG<br>GAGUAUUGUGAUAGAAUUAGAAG<br>AGAGG	Cloned into pET3a BglII-XhoI<br>In vitro transcription<br>MonoQ
model-SD (short)	28	5'-OH-<br>AUUUGGAGGUGAUUUAAAUGCCAA<br>AG	Dharmacon
Ss-aEF1A-like (short)	24	5'-OH-<br>UAAAGUGAUUUGAUGUAAUGAAAU<br>UACC	Eurogentec
Ss-MAP (short)	15	5'-PPP-AUGACUGAGGAUGAA	Eurogentec
Ss-aIF2β (short)	15	5'-PPP-GUGAGUUCAGAAAAA	In vitro transcription. MonoQ

Supplementary Table 1: model mRNAs used in the toeprinting experiments and cryo-EM studies
The Shine-Dalgarno sequence complementary to the anti-SD sequence is colored in red when present. The start codon and the base corresponding to the major toeprinting signal is colored in blue in long mRNAs. The sequences complementary to the toeprinting primers are shown in green. Model-SD, Ss-MAP, Ss-aEF1A-like mRNAs were chemically synthetized (Eurogentec). A 5' triphosphate group was chemically added to Ss-aEF1A-like mRNA (Eurogentec). Ss-aIF2β lmRNA was in vitro transcribed (see Methods). The violet bases in Ss-MAP short lmRNA indicate the pseudo-SD sequence located downstream from the start codon.

	DS1	DS1	DS2	DS2	DS2	DS3	DS3	DS4
**Data collection**	IC2-down	IC2-up	30S-HR	IC2-down	IC2-up	IC2-down	IC2-up	IC2-down
PDB ID	9FRK	9FRL	9FHL	9FRA	9FSF	9FY0	9FS6	9FS8
EMDB ID	50716	50717	50445	50709	50727	50854	50724	50725
Microscope	ESRF	ESRF	TFS Krios Pasteur	TFS Krios Pasteur	TFS Krios Pasteur	TFS Krios Pasteur	TFS Krios Pasteur	Glacios 2 Pasteur
Camera	Gatan K3		Gatan K3			Falcon4i		Falcon4i
Magnification	130000		105000			165000		130000
Voltage (kV)	300		300			300		200
Electron exposure (e–/Å^2)	40		40			20		40
Defocus range (µm)	-2.9 to -0.9		-3 to -1			-2.6 to -0.8		-2.6 to -0.8
Pixel size (Å)	1.053		0.86			0.73		0.88
Initial particle (no.)	269 000	269 000	975 000	975 000	975 000	234 000	234 000	308 783
Final particle (no.)	28 000	38 000	766 000	62 000	108 000	43 000	49 000	156
Resolution (unmasked. Å)	4.03	3.73	2.87	3.01	3.33	4.09	4	4.2
Resolution (masked. Å)	3.02	3	2.52	2.86	2.85	2.92	2.92	3.72
FSC threshold	0.143	0.143	0.143	0.143	0.143	0.143	0.143	0.143
**Refinement**								
d FSC model (Å). threshold 0.5	3.1	3.4	2.6	3	2.9	2.9	2.9	3.9
Map sharpening B factor (Å^2)	-	-	-96.41	-10	-10	-	-	-
Model composition								
Non-hydrogen atoms	66394	65224	64047	66676	65783	66885	65634	66643
Protein residues	3937	3937	393	3938	3936	4026	4029	4028
Nucleotides	1593	1540	1460	1597	1552	1574	1520	1579
Ions and spermines	ZN:8 MG:59 SPM:36	ZN:8 MG:53 SPM:31	ZN:8 MG:59 SPM:44	ZN:8 MG:58 SPM:42	ZN:8 MG:64 SPM:44	ZN:9 MG:47 SPM:43	ZN:9 MG:57 SPM:39	ZN:9 MG:57 SPM:30
Water molecules	165	210	515	221	276	273	199	91
Average B factors (Å^2)								
Protein	104.9	80.5	31.8	98.3	94.8	83.2	86.8	148
Nucleic acid	106.8	106.8	30.8	112.8	101.9	77.8	75.9	149.7
Ligand	83.2	81.8	25.1	85.3	84.4	67.5	64.4	127
Water molecules	73.5	77.1	20.3	78.2	79.22	53.7	50.1	122.8
R.m.s. deviations								
Bond lengths (Å)	0.009	0.005	0.007	0.004	0.005	0.005	0.008	0.008
Bond angles (°)	0.983	0.813	0.918	0.627	0.64	0.683	0.857	0.925
**Validation**								
MolProbity score	2.3	2.3	2.3	2.2	2.2	2.2	2.2	2.28
Clashscore	19.38	18.17	13.7	12.4	13	16.2	16	23.87
Poor rotamers (%)	2	2.38	3.3	2.5	2.5	2	2.1	1.52
Ramachandran plot								
Favored (%)	95.74	95.72	95.92	96.23	95.97	95.78	95.76	96.06
Allowed (%)	4.23	4.26	4.02	3.74	4	4.17	4.21	3.94
Disallowed (%)	0.03	0.03	0.05	0	0.03	0.05	0.03	0
Correlation coefficients								
Mask CC	0.87	0.8	0.84	0.9	0.9	0.86	0.86	0.85
Volume CC	0.87	0.8	0.81	0.9	0.9	0.85	0.82	0.84

Supplementary Table 2: Cryo-EM data collection, refinement and validation statistics

RNA modification		Position(s) in *S. solfataricus* 16S rRNA	Position(s) in *P. abyssi* 16S rRNA	Position(s) in *T. kodakarensis* 16S rRNA	Position(s) in H. Sapiens 18S rRNA
**ac^4C**	N4-acetylcytidine	1466,1467, 1477*,1478*	17,53,286,303, 319,379,394, 479,511,546, 590,626,636, 703,718,731, 751,828,839, 848,851,868, 957,1028, 1147,1233, 1239,1479	5,41,136,298,37 3,458,501,525, 569,605,697,730 ,818,827,847, 1007,1217,1459	1842
**Am**	2'-O-methyladenosine	494*	373	352	27,99,159,166,468,484,51, 576,590,668,1031,1383,16 78
**Cm**	2'-O-methylcytidine	246,313,481,1060, 1366	129,846,1036,1040 ,1376	190,296,535,752,825, 1354,1361	174,462,517,621,797,1272, 1391,1440,1703
**Gm**	2'-O-methylguanosine	337,399,672,927*, 1018*,1061,1194	467,471,519,657, 680,873,913,934, 1069	138,246,320,381,346,350, 446,450,498,532,636,659, 771,852,876,892,913,994, 1025,1026,1107,1265,1275, 1434	436,505,601,644,683,867,1 328,1447,1490
**m^5C**	5-methylcytidine	1368	535,693,875,1025, 1202,1374,1496, 1498,1505	457,464,672,854,942, 1004,1276,1374,1476, 1478	-
**m^6A**	N6-methyladenosine	1457*	1469	1449	1832
**$m^{6,6}A$**	N6.N6-dimethyladenosine	1475*	1487,1488	1467,1468	1850,1851
**Um**	2'-O-methyluridine	52,875*,1032,1344*	20,64,774,787,830, 1177,1380	8,52,240,304,479,1358	116,121,172,354,428,627,7 99,1288,1442,1668,1804
**$m^1acp^3\psi$**	1-methyl-3-α-amino-α-carboxyl-propyl pseudouridine	930*			1248

Supplementary Table 3 : **Modified residues localized in 16S rRNA sequence of *S. solfataricus* and compared to *P. abyssi, T. kodakarensis and H. sapiens.***

The name of the rRNA modification and its position in 16S rRNA sequences are indicated. The modifications colored in red are observed in the three archaea and in *H. sapiens* 18S rRNA. The asterisk indicates that the presence of a post-transcriptional modification at this position was observed in[20]. Reference structures are those of *P. abyssi* 16S rRNA (7ZHG)[21], *T. kodakarensis* (6TH6)[22], H. sapiens (8QOI)[23]. Note that $m^1acp^3\psi$ and ac^4C were not observed in bacterial 16S rRNA.

Name	Sequence
RT-m^{1}acp$^3\psi$930	5’-GCAAGGTCGTTAGCCTGGCCG
RT-m^{6,6}A1475-A1476	5’-GGAGGTGATCCAGCC
RT-ac4C1477-1478	5’-GATCCAGCCGCAGGTTCC
RT-ac4C1466-1467	5’-GGAGGTGATCCAGCC
RT-mapping5’-16S rRNA (also used to map 3’ end of 16S rRNA using ligated 16S rRNA)	5’-GCCACGTGTTACTCAGCCGTC
Cloning DNA-16S-forward	5’-TCGCCGGGCCCCTAAGCCCGGG
Cloning DNA-16S-backward	5’-TCTGTGAAAGGGAACCAAGGG
Amplification-Xho1 (also used for RT-PCR with ligated 16S RNA)	5’-CCGCAGCCCCATCTCGAGGCGCTCATGAGCGGG
Amplification BamH1	5’-GGGACAGCGGGATCCCGACCCCGAAAGGGGAAG
Toeprint primer Ss-MAP	5’-CCTTAGCACTAGCTTTAACGTC
Toeprint primer Ss-aEF1A-like	5’-GCTAATTCTTGAAGACGATCCGG
Toeprint primer Ss-aIF2β	5’-CCTATATTGAGTATTATCATATTAGGC
Toeprint primer model-SD	5’-TCGAACTTCTTGATGATGGTCTC
Cloning eS26-forward	5’-GGGTGTACATATGCCAAAGAAGAGAGAG
Cloning eS26-backward	5’-GATCGAGCGGCCGCTCTTAAACGAGCTTTTTG
Cloning Ss-aIF2β-forward (toeprint)	5’-AGACAGATCTTAATACGACTCACTATAGTGAGTTCAGAAAAAGAATACG
Cloning Ss-aIF2β-backward (for toeprint)	5’- TCGACTCGAGCCTCTCTTCTAATTCTATCACAATACTCC
Cloning Ss-aEF1A-like forward (for toeprint)	5’-AGACAGATCTTAATACGACTCACTATAGTTAAAGTGATTTGATGTAATGTAATGAAATTACC
Cloning Ss-aEF1A-like -backward (for toeprint)	5’- TCGACTCGAGCGCCTTCATCACTTACACCTATACG
Cloning Ss-MAP forward (for toeprint)	5’-AGAATCTAGACTCAGTCATCTGATGAGTCCGTGAGGACGAAACGGTACCCGGTACCGTCATGACTGAGGATGAACTC
Cloning Ss- MAP backward (for toeprint)	5’- TCGACTCGAGGGAAATGATGGAAACGCTTTATTTTC
T7 Promoter	5’-TAATACGACTCACTATA
Transcription Ss-aIF2β (Cryo-EM)	5’-TTTTTCTGAACTCACTATAGTGAGTCGTATTA

Supplementary Table 4: Oligonucleotides used in this study

Compounds	Elemental composition (MH^+)	MH^+ (*m/z*)	BH^+ (*m/z*)	$2MH^+$ (*m/z*)	tr (min)
C	$C_9H_{14}N_3O_5$	244.0928	112.050	487.17	1.3
m^5C	$C_{10}H_{16}N_3O_5$	258.1084	126.066	-	1.6
Cm	$C_{10}H_{16}N_3O_5$	258.1084	112.050	515.2091	2.2
U	$C_9H_{13}N_2O_6$	245.0768	113.035	489.1460	2.4
A	$C_{10}H_{14}N_5O_4$	268.1040	136.061	-	3.0
G	$C_{10}H_{14}N_5O_5$	284.0989	152.056	567.1901	3.6
Am	$C_{11}H_{16}N_5O_4$	282.1196	136.061	-	4.3
Gm	$C_{11}H_{16}N_5O_5$	298.1146	152.056	595.2227	4.8
mG	$C_{11}H_{16}N_5O_5$	298.1146	166.07		*4.9*
m^6A	$C_{11}H_{16}N_5O_4$	282.1196	150.077	-	4.9
ac^4C	$C_{11}H_{16}N_3O_6$	286.1033	154.061	-	5.2
$m^{6,6}A$	$C_{12}H_{18}N_5O_4$	296.1353	164.093	-	7

Supplementary Table 5: Detection of nucleosides in *S. solfataricus* 16S rRNA.
For each detected nucleoside. the retention time is indicated (See Methods). The measured molecular mass is indicated for the MH^+ nucleoside and for the BH^+ ion (nucleoside fragment ion derived from the base moiety). *Low intensity; nd : not detected. Gm was detected but not localized in the cryo-EM maps. The presence of multimers (2MH+) reveals higher concentration. Only the MH^+ nucleoside for $m_1acp^3\psi$ was detected.

type	number
RNA nucleotides	36
RNA atoms	1171
Mg^{2+} ions	3
exp. water	36
total water	5154
total atoms	16636

Supplementary Table 6: Description of the system simulated by MD.
The number of RNA nucleotides and atoms, Mg^{2+} ions, water molecules (experimentally placed and total), as well as the total number of atoms are indicated.

region	Volume (Å^3)	gO			gH			Esw-dens (kcal/mol/Å^3)			Eww-dens (kcal/mol/Å^3)		
A3P		A	B	AB	A	B	AB	A	B	AB	A	B	AB
1	4050.0	0.60	0.61	0.60	0.65	0.65	0.65	-0.39	-0.40	-0.39	-0.04	-0.04	-0.04
	(0.0)	(0.00)	(0.00)	(0.00)	(0.00)	(0.00)	(0.00)	(0.00)	(0.00)	(0.00)	(0.00)	(0.00)	(0.00)
2	250.5	0.61	0.65	0.63	0.69	0.70	0.70	-0.24	-0.26	-0.25	-0.10	-0.10	-0.10
	(0.0)	(0.00)	(0.00)	(0.00)	(0.00)	(0.00)	(0.00)	(0.00)	(0.00)	(0.00)	(0.00)	(0.00)	(0.00)
3	110.6	1.25	1.28	1.26	1.19	1.18	1.18	-0.50	-0.51	-0.51	-0.20	-0.20	-0.20
	(0.0)	(0.00)	(0.00)	(0.00)	(0.00)	(0.00)	(0.00)	(0.00)	(0.00)	(0.00)	(0.00)	(0.00)	(0.00)
4	2.4	3.80	3.70	3.75	2.06	1.92	1.99	-1.55	-1.61	-1.58	-0.63	-0.57	-0.60
	(0.0)	(0.01)	(0.01)	(0.00)	(0.02)	(0.00)	(0.01)	(0.01)	(0.01)	(0.01)	(0.00)	(0.00)	(0.00)

Supplementary Table 7: Water properties averaged over different regions, calculated with GIST, from 100 ns MD simulations *including* experimentally placed water molecules.
Properties presented are water oxygen (gO) and hydrogen (gH) densities relative to bulk water density, solute-water (Esw) and water-water (Eww) interaction energy densities. Regions considered are: 1) the whole GIST grid (gray edges in Supplementary Figure 16B), 2) the region above G889 nucleobase of 16S rRNA (gray edges in Supplementary Figure 16C), 3) a subset of previous region (black edges in Supplementary Figure 16C), 4) the five experimentally placed water oxygens above G889 nucleobase (red spheres in Supplementary Figure 16C). Two conformations (A and B) of 5'-terminal triphosphate nucleotide (A3P) of mRNA were considered, as well as their average densities (AB). Values indicated are averages over three replicates, obtained with different initial velocities. On each subsequent line, the standard deviations over the replicates are given in parentheses.

region	Volume (Å^3)	gO			gH			Esw-dens (kcal/mol/Å^3)			Eww-dens (kcal/mol/Å^3)		
A3P		A	B	AB	A	B	AB	A	B	AB	A	B	AB
1	4050.0	0.60	0.61	0.60	0.65	0.65	0.65	-0.39	-0.40	-0.39	-0.04	-0.04	-0.04
	(0.0)	(0.00)	(0.00)	(0.00)	(0.00)	(0.00)	(0.00)	(0.00)	(0.00)	(0.00)	(0.00)	(0.00)	(0.00)
2	250.5	0.61	0.65	0.63	0.69	0.70	0.70	-0.24	-0.26	-0.25	-0.10	-0.10	-0.10
	(0.0)	(0.00)	(0.00)	(0.00)	(0.00)	(0.00)	(0.00)	(0.00)	(0.00)	(0.00)	(0.00)	(0.00)	(0.00)
3	110.6	1.25	1.28	1.26	1.19	1.18	1.18	-0.50	-0.51	-0.51	-0.20	-0.20	-0.20
	(0.0)	(0.00)	(0.00)	(0.00)	(0.00)	(0.00)	(0.00)	(0.00)	(0.00)	(0.00)	(0.00)	(0.00)	(0.00)
4	2.4	3.79	3.69	3.74	2.05	1.92	1.99	-1.55	-1.63	-1.59	-0.63	-0.56	-0.59
	(0.0)	(0.03)	(0.02)	(0.01)	(0.01)	(0.02)	(0.01)	(0.02)	(0.01)	(0.01)	(0.00)	(0.01)	(0.00)

Supplementary Table 8: Water properties averaged over different regions, calculated with GIST, from 100 ns MD simulations *not including* experimentally placed water molecules.
Same details as Supplementary Table 7.

region	Volume (Å^3)	gO			gH			Esw-dens (kcal/mol/Å^3)			Eww-dens (kcal/mol/Å^3)		
A3P		A	B	AB	A	B	AB	A	B	AB	A	B	AB
1	4050.0	0.60	0.61	0.60	0.65	0.65	0.65	-0.39	-0.40	-0.39	-0.04	-0.04	-0.04
	(0.0)	(0.00)	(0.00)	(0.00)	(0.00)	(0.00)	(0.00)	(0.00)	(0.00)	(0.00)	(0.00)	(0.00)	(0.00)
2	250.5	0.61	0.65	0.63	0.69	0.70	0.70	-0.24	-0.26	-0.25	-0.10	-0.10	-0.10
	(0.0)	(0.00)	(0.00)	(0.00)	(0.00)	(0.00)	(0.00)	(0.00)	(0.00)	(0.00)	(0.00)	(0.00)	(0.00)
3	110.6	1.25	1.28	1.26	1.19	1.18	1.18	-0.50	-0.51	-0.51	-0.20	-0.20	-0.20
	(0.0)	(0.00)	(0.00)	(0.00)	(0.00)	(0.00)	(0.00)	(0.00)	(0.00)	(0.00)	(0.00)	(0.00)	(0.00)
4	2.4	3.81	3.67	3.74	2.05	1.93	1.99	-1.56	-1.60	-1.58	-0.63	-0.56	-0.60
	(0.0)	(0.03)	(0.03)	(0.01)	(0.02)	(0.01)	(0.01)	(0.00)	(0.02)	(0.01)	(0.01)	(0.00)	(0.00)

Supplementary Table 9: Water properties averaged over different regions, calculated with GIST, from 75 ns MD simulations *including* experimentally placed water molecules.
Same details as Supplementary Table 7.

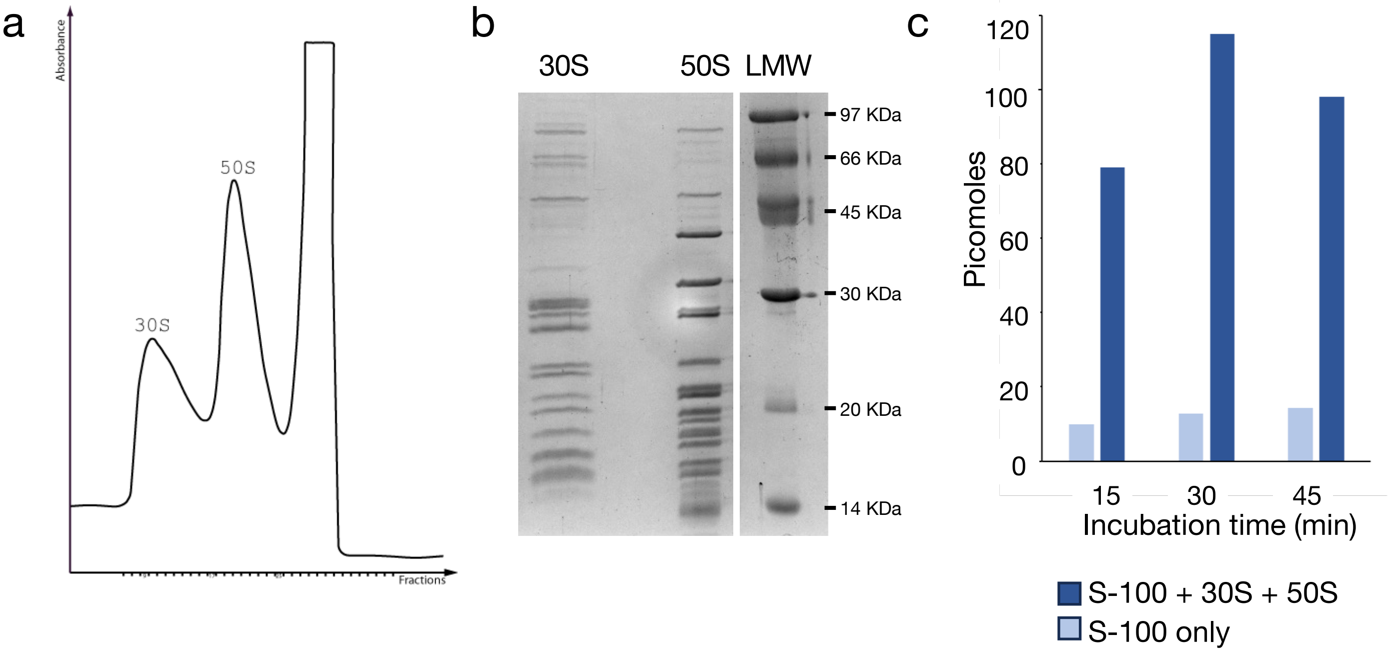

Supplementary Figure 1: Preparation of *S. solfataricus* ribosomal subunits

a. Sucrose gradient (10-30%) profile of the ribosomal subunits in buffer A.
b. SDS-PAGE analysis 30S and 50S preparations. Lane 1: 30S, lane 2: 50S. This 30S preparation was analyzed by mass spectrometry.
c. In vitro translation assay. In vitro poly-U translation conditions were adapted from[24,25], see Methods. The graph shows the amount of [^{3}H]Phe polymerized after the indicated times of incubation, in the presence of S-100 fraction only (light blue bars) or in the presence of S-100 plus purified 30S and 50S (dark blue bars). The experiment shows that the purified ribosomal subunits are active in poly-Phe synthesis, being able to polymerize 1 pmol phe/pmol 50S particles at 60°C, in reasonable agreement with[25].

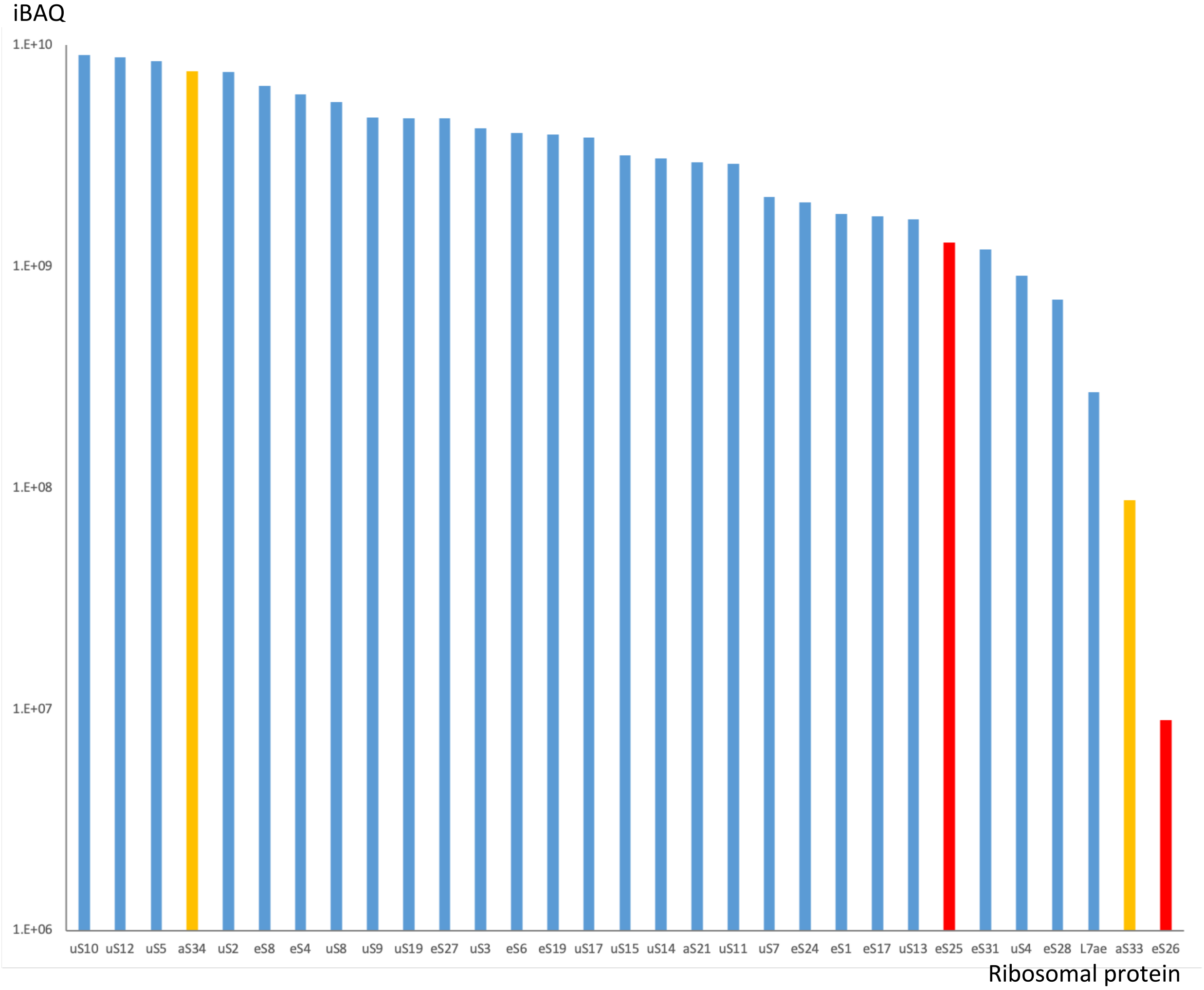

Supplementary Figure 2: Ribosomal proteins identified by nanoLC-MS/MS analysis

Mass spectrometry analysis (see methods) was performed on the 30S sample shown in Supplementary Figure 1B. Data processing was performed with MaxQuant software against a database containing the *S. solfataricus* P2/DSM1617 proteome (Uniprot), the eS26 sequence (tr|A0A0E3MCW1|A0A0E3MCW1_SACSO;Q97ZR1) and that of D0KTI0_SACS9 (named here aS33) which were not in the *S. solfataricus* library. iBAQ intensities are plotted in a log scale for each ribosomal protein. aS33 and aS34 are shown with orange bars. eS25 and eS26 are shown with red bars. eS30, observed in our 30S cryo-EM structures, was not detected in this nanoLC-MS/MS analysis likely because of its small size and high-content of lysine and arginine residues (9 K, 7 R out of 51 residues, leading only to three potentially detectable peptides).

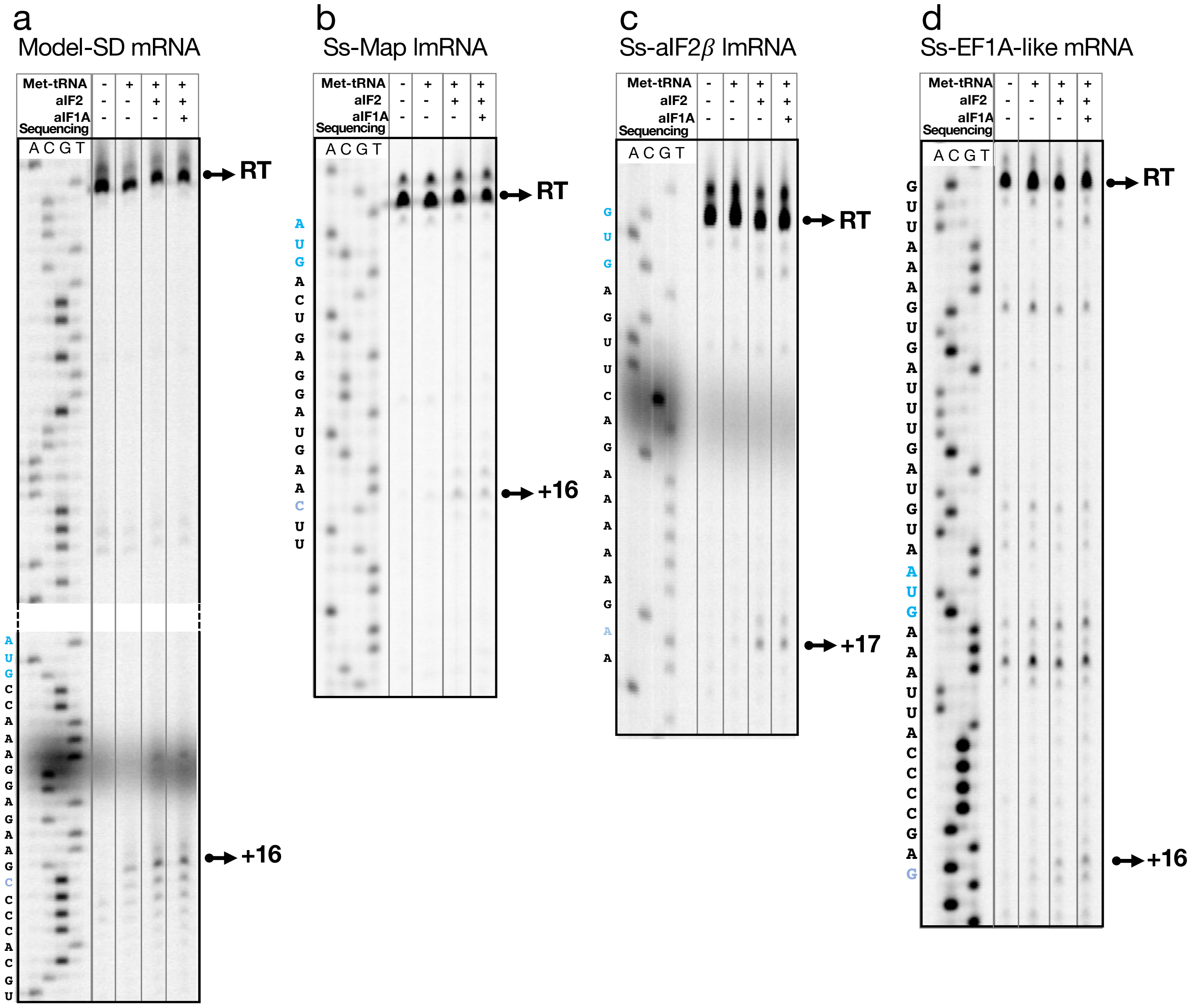

Supplementary Figure 3: Examples of toeprinting analysis of 30S complexes assembled on various mRNAs in the presence of the indicated initiation factor and of Met-tRNA$_i^{Met}$

a. Typical toeprinting experiment using model-SD mRNA. The mRNA sequence complementary to the DNA sequence is shown on the left of the gel image. The positions of the start codon and of the main toeprinting signals are indicated in blue. Full-length cDNA is marked RT, Lane 1: toeprinting signal for a 30S:mRNA complex, lane 2: 30S:mRNA:Met-tRNA$_i^{Met}$, lane 3: 30S:mRNA-Met-tRNA$_i^{Met}$:aIF2, lane 4: 30S:mRNA-Met-tRNA$_i^{Met}$:aIF2:aIF1A (see also Figure 1A).
b. Same as panel a but using the Ss-MAP lmRNA.
c. Same as panel a but using the Ss-aIF2β lmRNA.
d. Same as panel a but using the Ss-EF1A-like mRNA. Uncropped gels are shown in Supplementary data files.

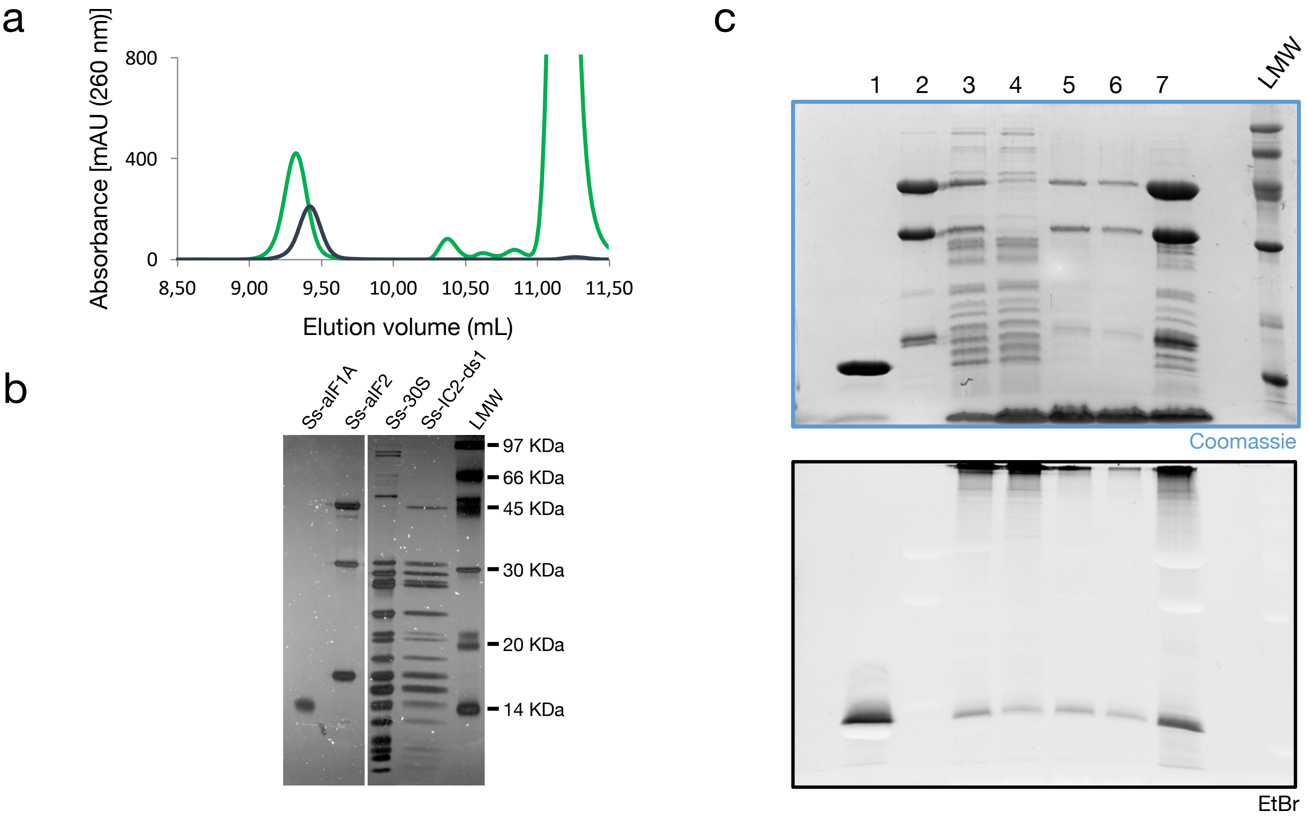

Supplementary Figure 4: Preparation of initiation complexes

a. Bio-Agilent Sec5 chromatograms for Ss-30S (black) and model-SD mRNA initiation complex (IC) used to collect dataset 1 (green).
b. SDS-PAGE analysis of Ss-aIF1A, Ss-aIF2, Ss-30S, and Ss-IC2-DS1 purified by size exclusion chromatography. The gel was stained using SYPRO rubis (Thermo Fisher).
c. SDS-PAGE analysis of affinity purification steps of IC used to collect dataset 2. The gel was stained with Coomassie blue (up) and ethidium bromide (bottom) to reveal the initiator tRNA. Lane 1: mixture of Ss-aIF1A and Met-tRNA, lane 2: Ss-aIF2, lane 3: mixture of Ss-30S + Ss-MAP-lmRNA + Ss-aIF1A + Ss-aIF2 + Met-tRNA$_i^{Met}$, lane 4: Flow-through fraction, lanes 5-6: elution fractions, lane 7: concentrated sample.

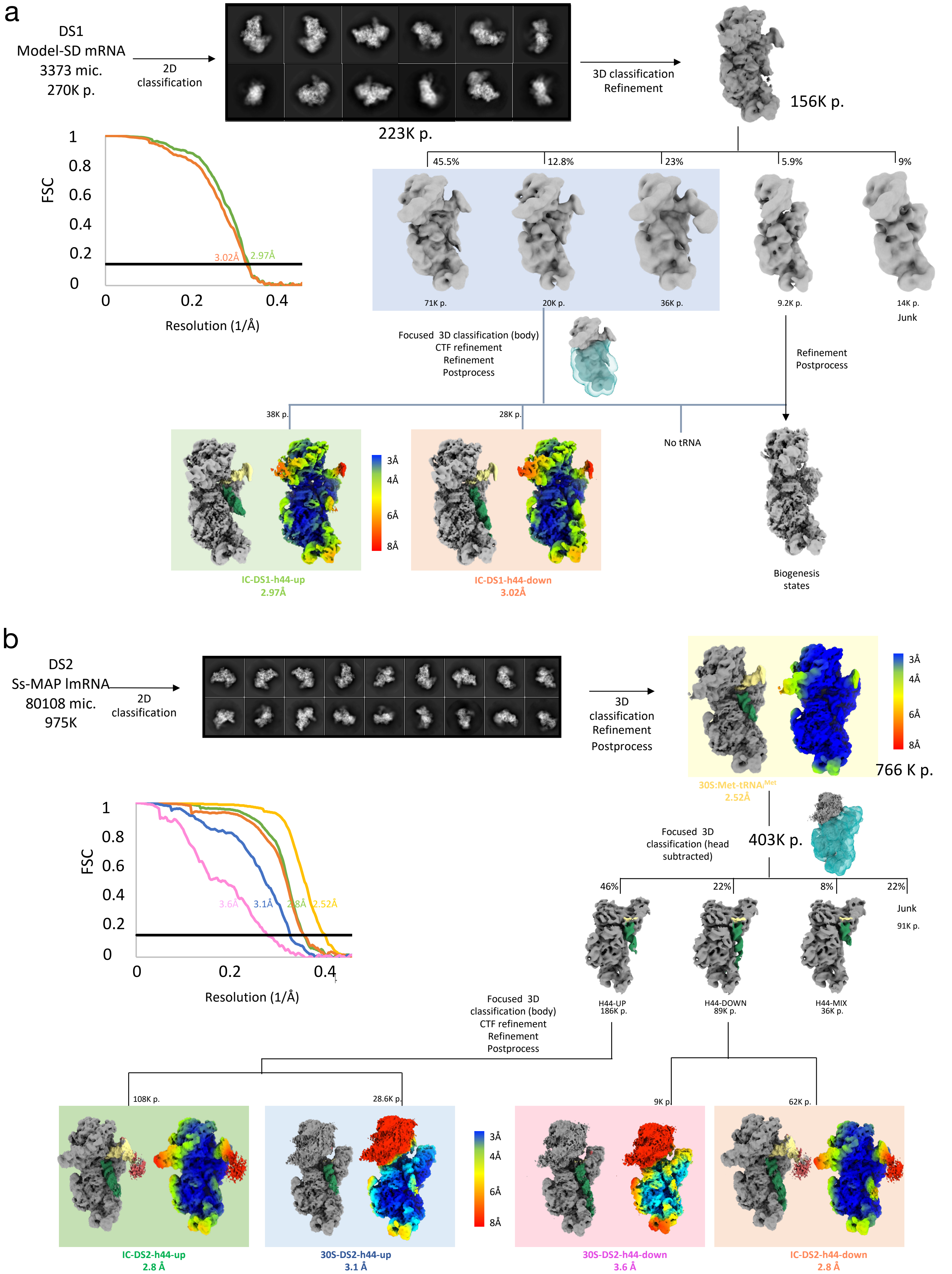
a
DS1
Model-SD mRNA
3373 mic.
270K p.
2D classification
223K p.
3D classification
Refinement
156K p.
FSC
Resolution (1/Å)
3.02Å
2.97Å
45.5%
12.8%
23%
5.9%
9%
71K p.
20K p.
36K p.
9.2K p.
14K p.
Junk
Focused 3D classification (body)
CTF refinement
Refinement
Postprocess
Refinement
Postprocess
38K p.
28K p.
No tRNA
3Å
4Å
6Å
8Å
IC-DS1-h44-up
2.97Å
IC-DS1-h44-down
3.02Å
Biogenesis states
b
DS2
Ss-MAP lmRNA
80108 mic.
975K
2D classification
3D classification
Refinement
Postprocess
766 K p.
30S:Met-tRNAi^Met
2.52Å
FSC
Resolution (1/Å)
3.6Å
3.1Å
2.8Å
2.52Å
Focused 3D classification (head subtracted)
403K p.
46%
22%
8%
22%
Junk
91K p.
H44-UP
186K p.
H44-DOWN
89K p.
H44-MIX
36K p.
Focused 3D classification (body)
CTF refinement
Refinement
Postprocess
108K p.
28.6K p.
9K p.
62K p.
IC-DS2-h44-up
2.8 Å
30S-DS2-h44-up
3.1 Å
30S-DS2-h44-down
3.6 Å
IC-DS2-h44-down
2.8 Å

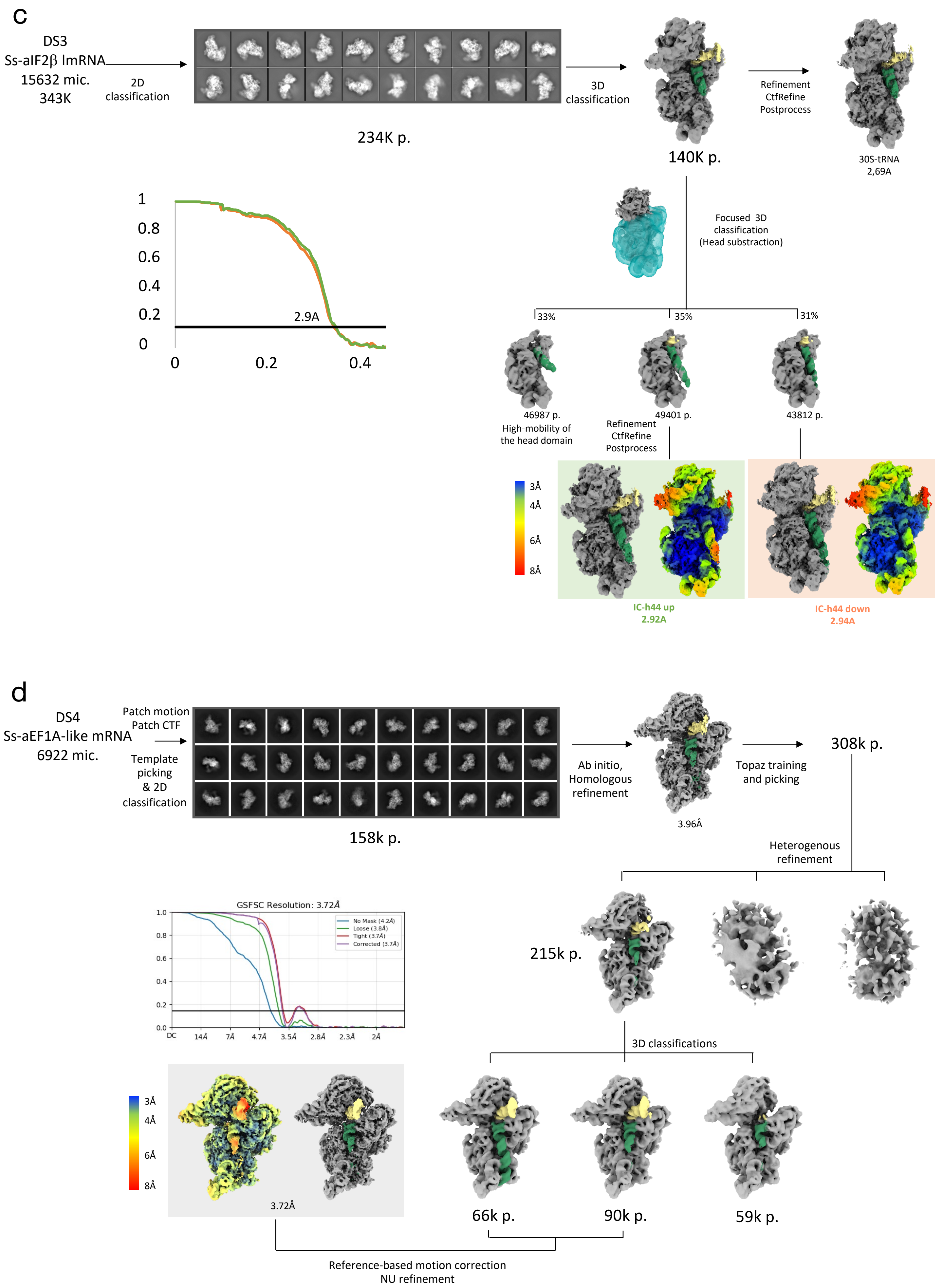
c
DS3
Ss-aIF2β lmRNA
15632 mic.
343K
2D classification
234K p.
3D classification
140K p.
Refinement
CtfRefine
Postprocess
30S-tRNA
2,69A
1
0.8
0.6
0.4
0.2
0
0
0.2
0.4
2.9A
Focused 3D classification
(Head substraction)
33%
35%
31%
46987 p.
High-mobility of the head domain
49401 p.
43812 p.
Refinement
CtfRefine
Postprocess
3Å
4Å
6Å
8Å
IC-h44 up
2.92A
IC-h44 down
2.94A
d
DS4
Ss-aEF1A-like mRNA
6922 mic.
Patch motion
Patch CTF
Template picking
& 2D classification
158k p.
Ab initio,
Homologous refinement
3.96Å
Topaz training and picking
308k p.
Heterogenous refinement
215k p.
GSFSC Resolution: 3.72Å
No Mask (4.2Å)
Loose (3.8Å)
Tight (3.7Å)
Corrected (3.7Å)
1.0
0.8
0.6
0.4
0.2
0.0
DC
14Å
7Å
4.7Å
3.5Å
2.8Å
2.3Å
2Å
3D classifications
3Å
4Å
6Å
8Å
3.72Å
66k p.
90k p.
59k p.
Reference-based motion correction
NU refinement

e

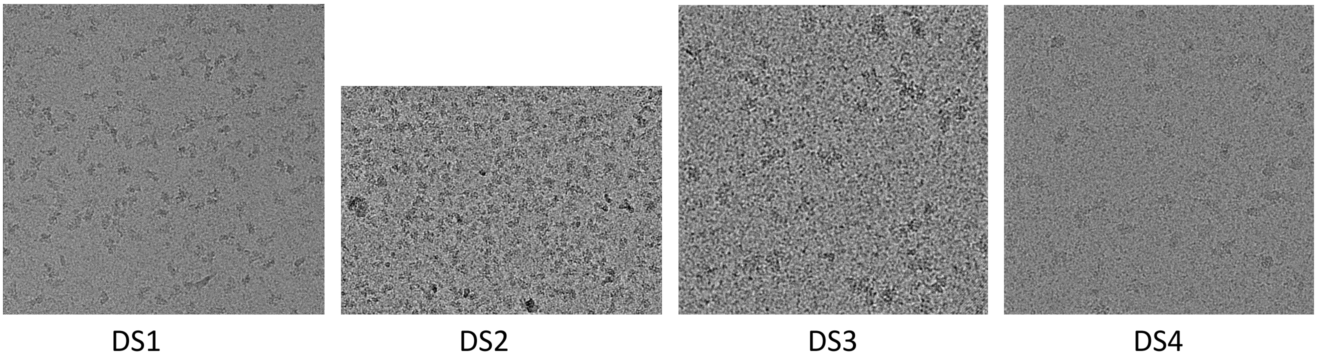

f

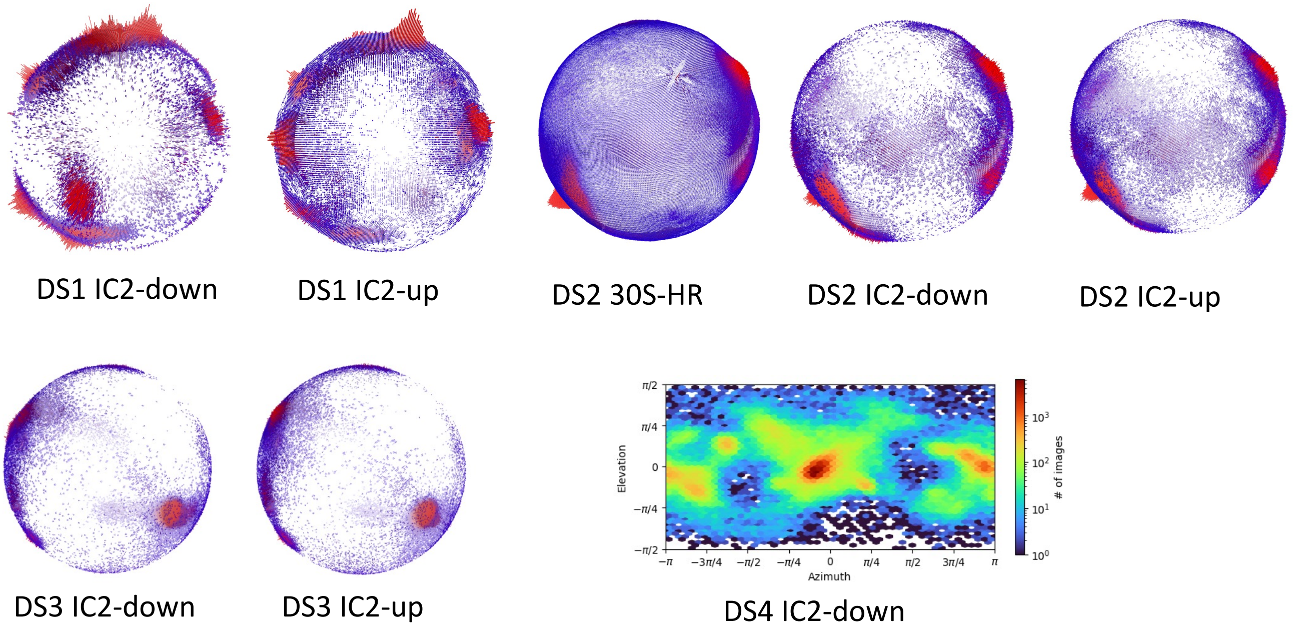

Supplementary Figure 5: Cryo-EM data processing

a. Flowcharts of cryo-EM data processing of dataset 1 (DS1) corresponding to an initiation complex assembled on the model-SD mRNA. Data processing was conducted in Relion[26]. Cryo-EM maps filtered and coloured by local resolution are shown. To sort the h44-up and –down conformations, focused 3D classification[27] was done with a mask containing the 30S body and the possible conformations of h44. This mask is shown as a blue transparent surface. Fourier shell correlation curves[28-30] were obtained by masking the two half maps and calculating the cross-correlation between the masked volumes. Resolution was estimated using the 0.143 cutoff criterion. The green curve is for the IC-DS1-h44-up complex and the orange curve is for the IC-DS1-h44-down complex.
b. Same analysis for dataset 2 (DS2). The IC complex is assembled on Ss-MAP lmRNA. To sort the h44-up and -down conformations, the density of the 30S head was first subtracted. The subtracted particles were then submitted to focused 3D classification with a mask encompassing the 30S body, the tRNA and the possible conformations of h44. Fourier shell correlation curves are as follows: yellow for 30S:Met-tRNA$_i^{Met}$, green for IC-DS2-h44-up, orange for IC-DS2-h44-down, blue for 30S-DS2-h44-up, violet for 30S-DS2-h44-down.
c. Same analysis for dataset 3 (DS3). The IC complex is assembled on Ss-aIF2β lmRNA. The strategy for sorting the h44-up and -down conformations was the same as for DS2. Fourier shell correlation curves are as follows: green for IC-DS3-h44-up, orange for IC-DS3-h44-down.
d. Data processing of dataset 4 (DS4). The IC complex is assembled on EF1A-like mRNA. Data processing was conducted in cryosparc 4.0[31].
e. Representative micrographs for the four datasets.
f. Angular distributions of particles used for each reconstruction (see Supplementary Table 2), in a 3D representation, as generated in Relion, or in a 2D representation, as generated in Cryosparc.

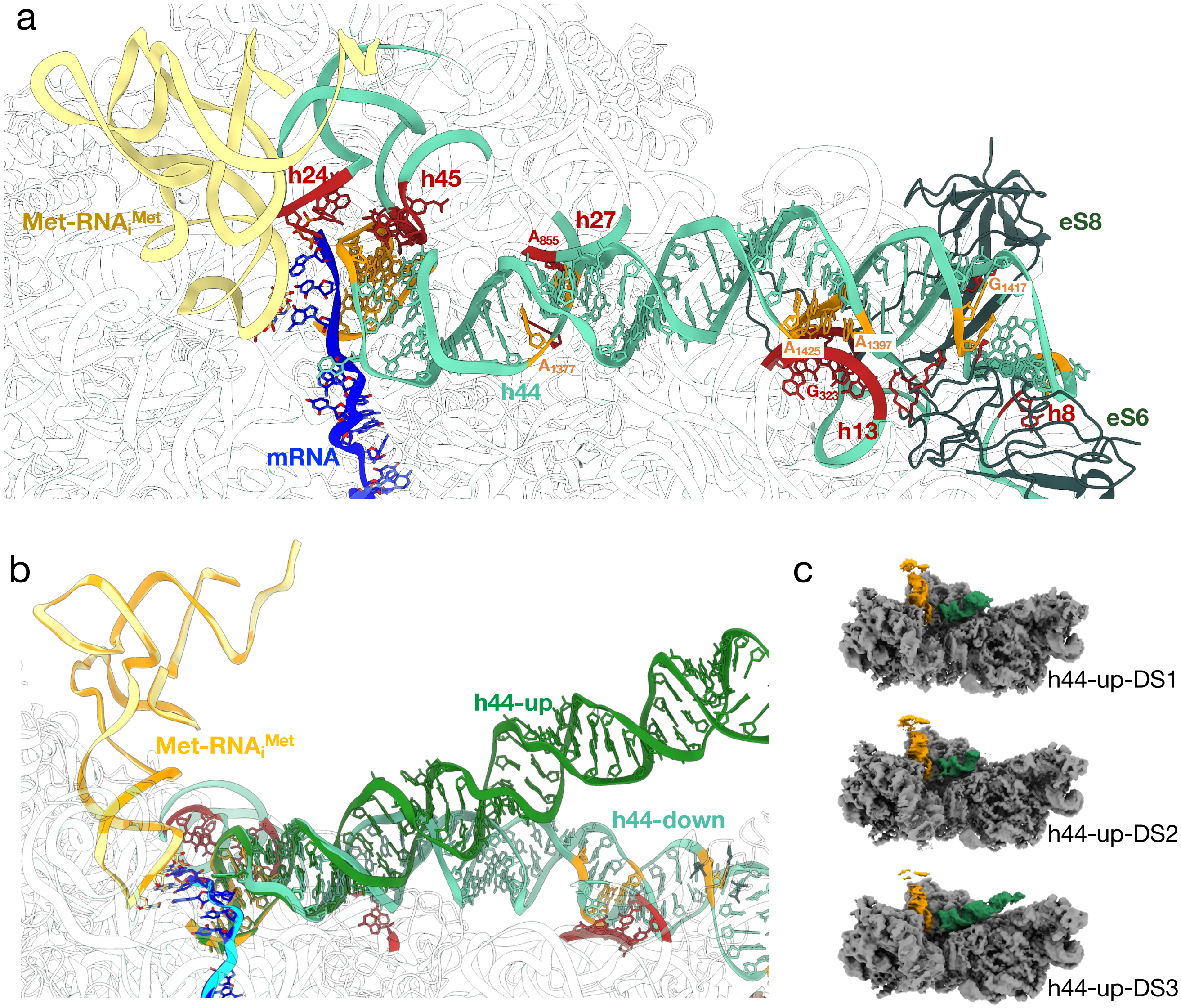

Supplementary Figure 6: Contacts between h44 and 30S

a. The upper part of h44 and residues involved in interactions with the body of the 30S are shown in orange. The mRNA is in dark blue and the initiator Met-tRNA$_i^{Met}$ is in yellow. The upper part of h44 interacts with h24 and h45 and forms tRNA binding sites. A1377 and C1379 interact with A872 and A855 in h27 bulge; A1397 and A1425 interact with the minor groove of h13 via A-minor interactions with residues G323 and C340. The last bulge of the h44 helix is packed onto h8 minor groove. Two ribosomal proteins interacts with h44, eS8 and eS6.
b. Superimposition of the h44-up and h44-down IC-DS2 cryo-EM structures. The h44-down structure is shown with the same color as in panel A. For the h44-up structure, h44 is in green, the tRNA is in orange and the mRNA is in cyan. The view shows that the conformation of the upper part of h44 is not changed in the up or in the down structures.
c. Cryo-EM maps for DS1, DS2 and DS3 showing h44-up positions (green) and the tRNA.

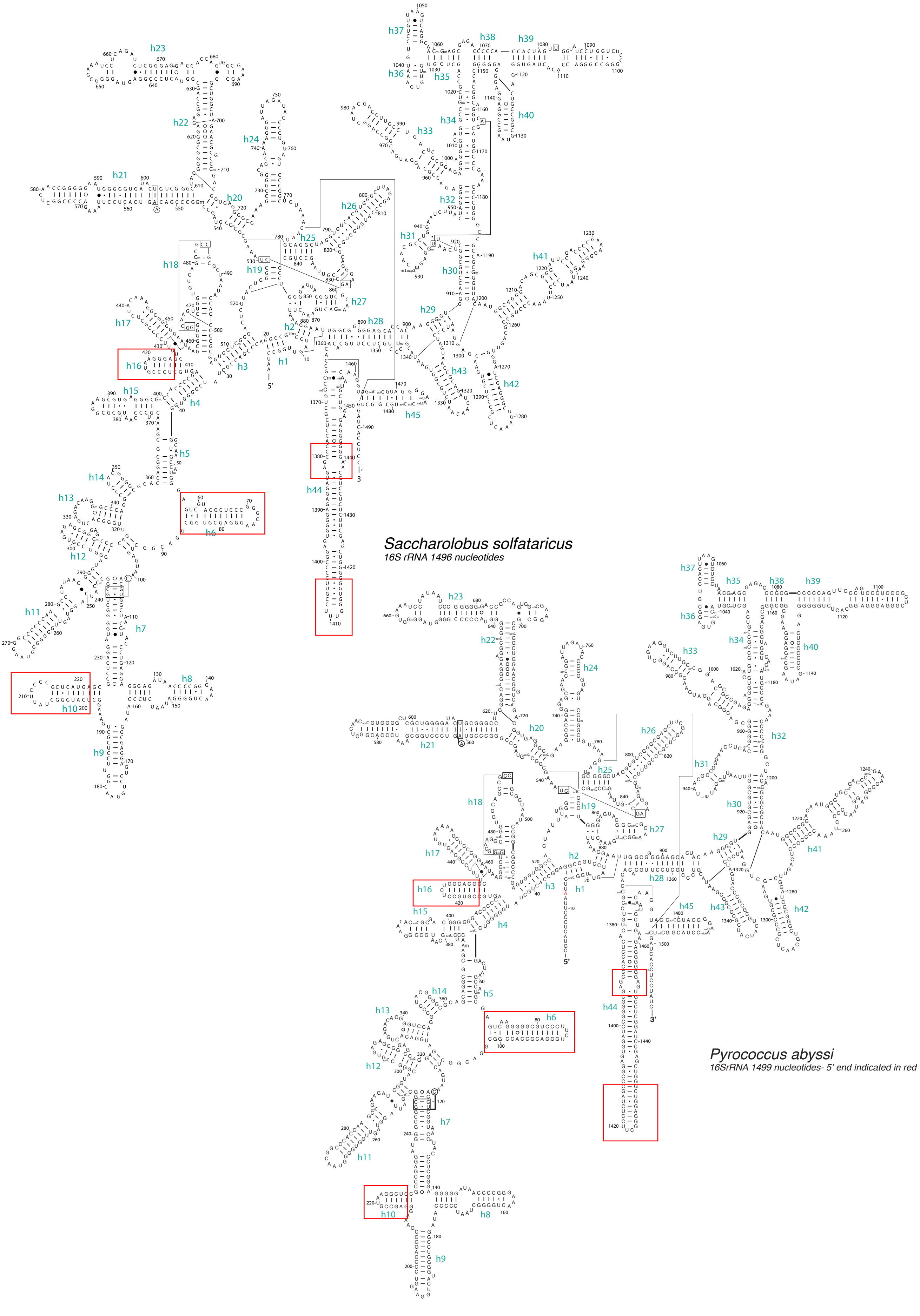

Supplementary Figure 7: Comparison of 16S rRNAs from S. *solfataricus* and *P. abyssi*

Secondary structure diagrams were retrieved from https://crw-site.chemistry.gatech.edu/ and updated according to the cryo-EM structures. The 5' end of *P. abyssi* 16S rRNA observed in the cryo-EM structure is indicated with a red letter.

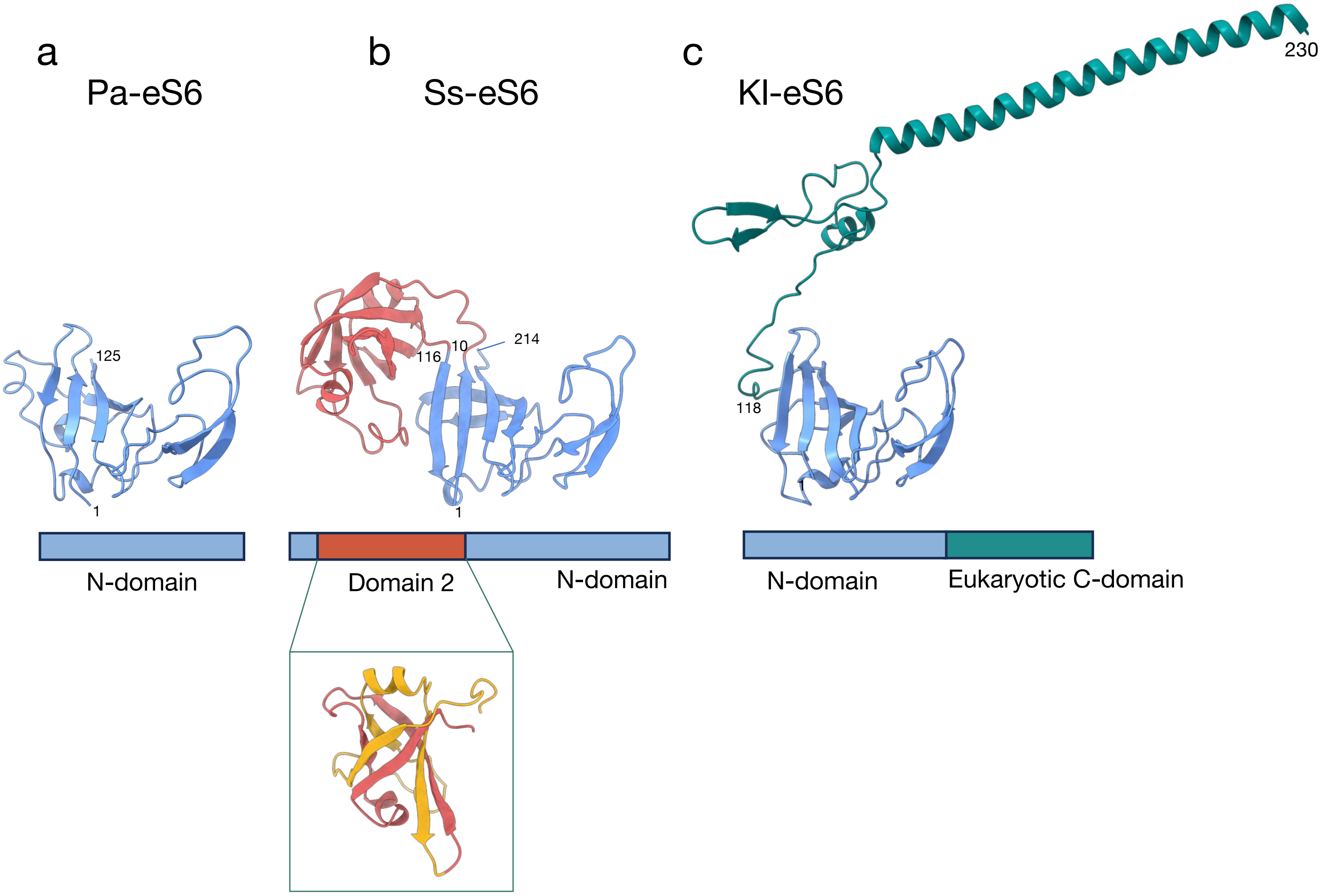

Supplementary Figure 8: Archaeal and eukaryotic eS6

a. *P. abyssi* eS6 (PDB 7ZHG)[21].

b. *S. solfataricus* eS6 (present study). A cartoon of *S. solfataricus* eS6 domain 2 showing the double-psi β-barrel made up of two pseudo-symmetric ββαβ units is also shown. eS6-domain 2 constructed from the cryo-EM map is superimposable on an AlphaFold model with an rmsd of 0.46 Å for 100 C-alpha atoms compared.

c. *K. lactis* eS6 (PDB 6FYX)[32].

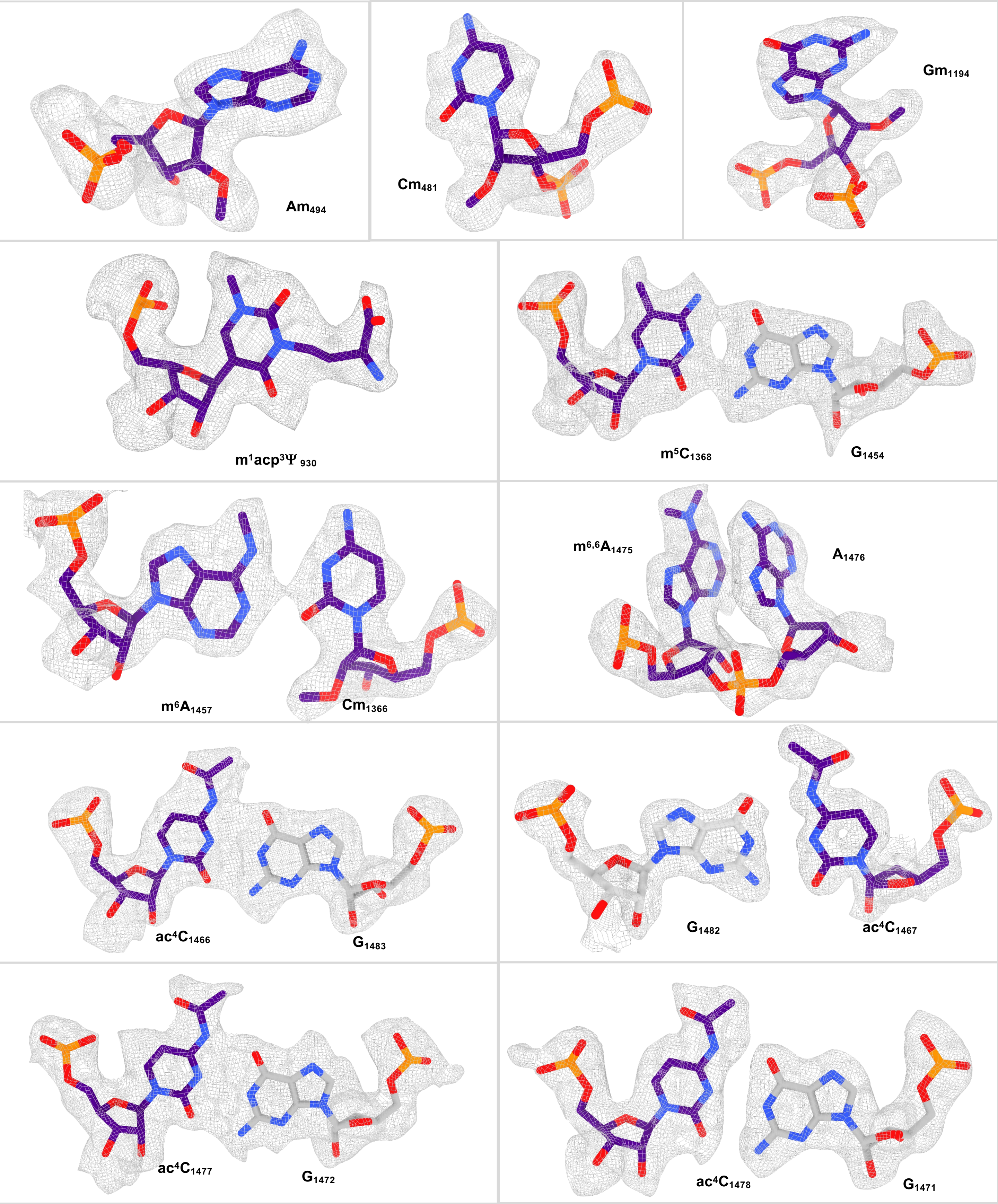

Am_{494}
Cm_{481}
Gm_{1194}
$m^1acp^3\Psi_{930}$
m^5C_{1368}
G_{1454}
m^6A_{1457}
Cm_{1366}
$m^{6,6}A_{1475}$
A_{1476}
ac^4C_{1466}
G_{1483}
G_{1482}
ac^4C_{1467}
ac^4C_{1477}
G_{1472}
ac^4C_{1478}
G_{1471}

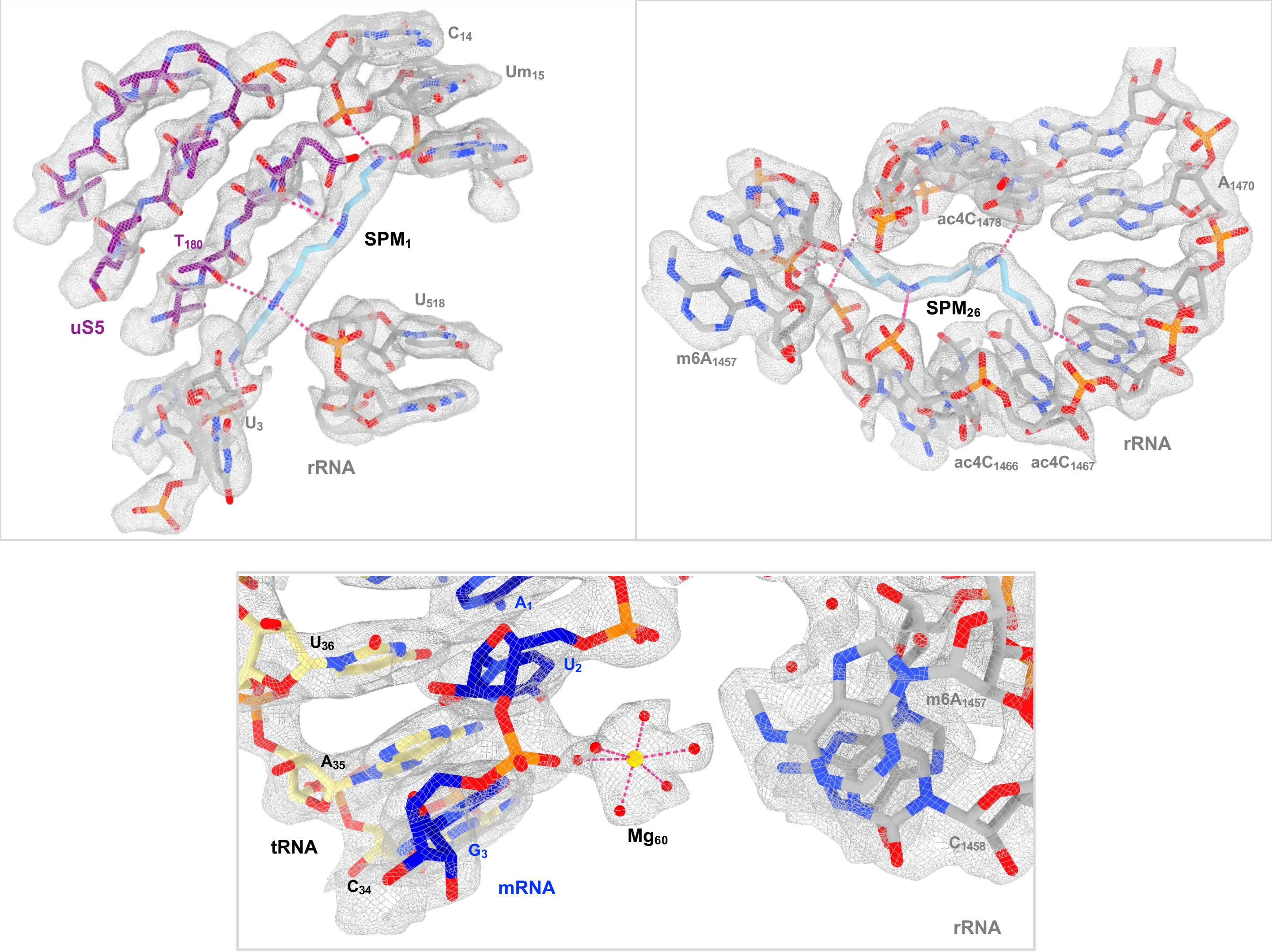

Supplementary Figure 9: Examples showing the quality of the cryo-EM map for rRNA modified nucleotides, spermine residues, magnesium ions

The 2.5 Å resolution cryo-EM map (DS2) is shown using the zone command in ChimeraX.

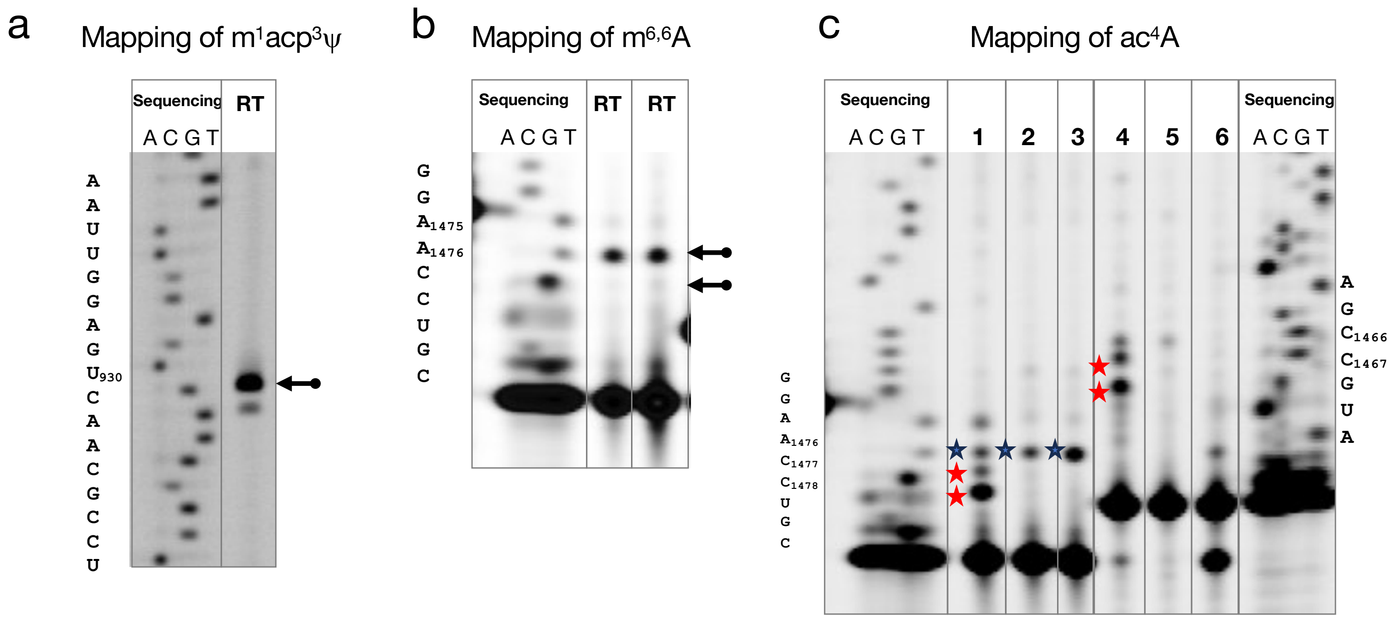

Supplementary Fig. 10: Primer extension analysis of 16S rRNA

a. Mapping of the m^{1}acp$^{3}\psi$ modification expected at position 930. The arrow indicates the strong arrest of the reverse transcriptase one nucleotide before the expected modification. The weak arrest observed one nucleotide below could be due to the large steric hindrance of this modification.

b. Mapping of the modifications m^{6,6} expected at positions A1475 and A1476. The arrow indicates the strong arrest of the reverse transcriptase one nucleotide before the expected modification. The experiment shows the presence of a modification at position A1475. However, no convincing arrest signal showing the presence of an m^{6,6} modification at position A1476 was observed (see also Supplementary Figure 9).

c. Primer extension analysis of 16S rRNA to map ac^{4}C residues. Lanes 1 and 4, 16S rRNA treated with NaBH4 (100mM, 37°C, 1h as described in[33]), lanes 2 and 5 control 16S rRNA (37°C, 1h), lanes 3 and 6, intact 16S rRNA. The RT stops are indicated to the left of the bands with stars. Lanes 2 and 3 indicate the RT stop due to the presence of a dimethyl adenosine at position 1475. This stop occurs even in the presence of NaBH4 treatment. Lane 1 shows two additional arrests, as compared to lanes 2 and 3, indicative of ac4C in positions 1477 and 1478 (see also supplementary Figure 9). Lane 4 indicates two additional arrests (red stars), as compared to lanes 5 and 6, indicative of ac4C in positions 1466 and 1467 (see also supplementary Figure 9). Uncropped gels are shown in Supplementary data files. Sequences of the RT primers are given in Supplementary Table 4.

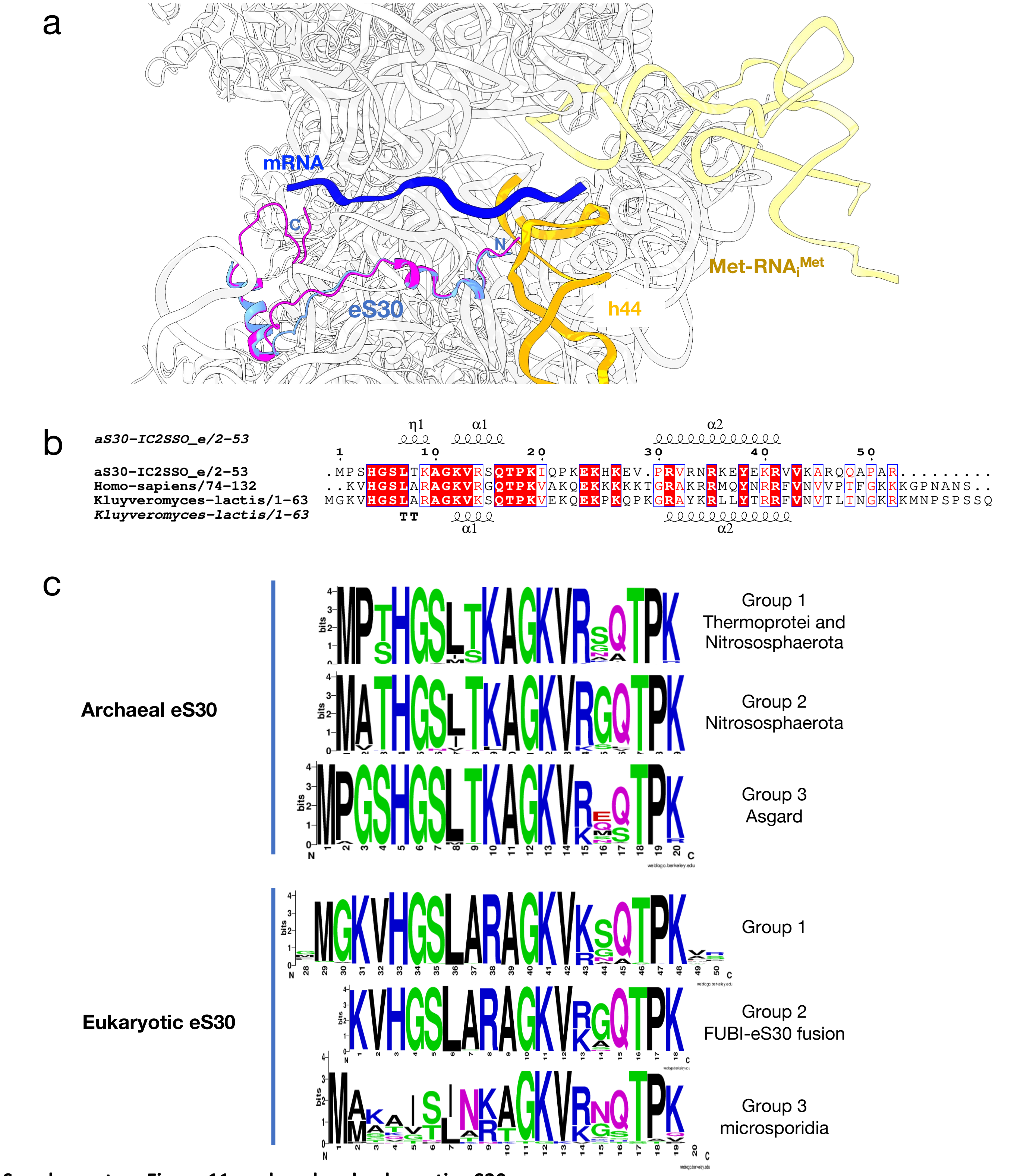

Supplementary Figure 11: archaeal and eukaryotic eS30

a. Cartoon view of IC-DS2 with Ss-eS30 colored in cornflower blue. eS30 from superimposed human ribosome (8G5Z)[34] or (8QOI)[23] is colored in magenta. The view shows a remarkable conservation between archaeal and eukaryotic eS30 structures. Eukaryotic versions of eS30 have a longer C-terminal tail. Moreover, variability is observed at the N-terminal ends of the eukaryotic and archaeal proteins as shown in view b.

b. Archaeal and eukaryotic eS30 protein sequences were retrieved from Uniprot and aligned usingClustal Omega. The multi-sequence alignment was manually corrected using Jalview editor[35]. 703 archaeal sequences were aligned. The sequences corresponding to the first 19 amino acids were extracted. Three main groups were defined to calculate logos (https://weblogo.berkeley.edu/logo.cgi). Group 1 contains 521 sequences coming from Thermoprotei and Nitrososphaerota. Group 2 contains 84 sequences from Nitrososphaerota (all having MATH as first amino acids) with some Bathy-, Brock- and one Heimdall- archaeota. Group 3 contains 101 sequences from Asgard only (one exception for an unidentified thermococci) . Notably, Heimdallarchaeota have MAGSH instead of MPGSH. 2098 eukaryotic eS30 sequences manually curated were aligned. 829 sequences were identified as ubiquitin-like fusion protein FUBI-eS30. In these cases, the N-terminal extremity of eS30 obtained after cleavage of the FUBI-eS30 fusion protein was proposed to be the lysine of the KVHGS[36-38]. The remaining 1243 sequences corresponding to N-terminal regions were extracted and used to calculate a logo. The vast majority of sequences starts at position 29 of the multiple alignement. 25 sequences of microsporidia were considered separately. Collectively, this analysis shows that archaeal versions of eS30 have sequence specificities at the N-terminus as compared to eukaryotes. The archaeal version of eS30 that is closest to eukaryotes is that from Heimdallarchaeota. Sequence alignments are available as Supplementary data.

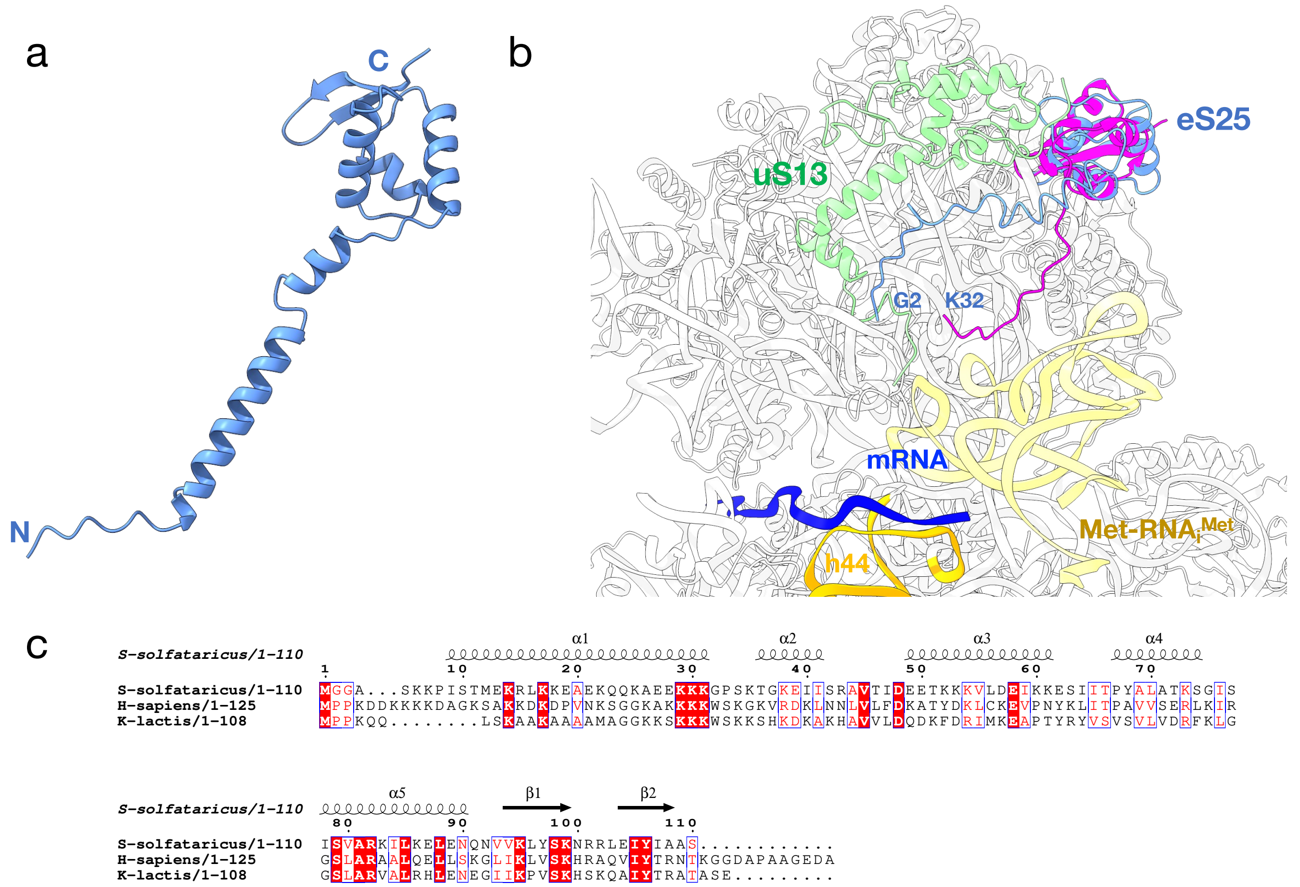

Supplementary Figure 12: **archaeal and eukaryotic eS25**

a. Ss-eS25 Alphafold2 model. The same prediction is available for eukaryotic and archaeal eS25.
b. Cartoon representation of IC-DS2 with Ss-eS25 colored in cornflower blue. Region 12 to 35 was only tentatively placed in a poorly defined part of the cryo-EM map. Human eS25 (PDB 8G5Z[34]) is colored in magenta. Residues 1 to 32 of human eS25 are not visible.
c. Structural alignment of *S. solfataricus* eS25 with the human and *K. lactis* proteins. Notably, sequence alignment of 250 archaeal versions of eS25 show that about 70% of the sequences (most TACK except some Acidilobales, Desulfurococcales and Fervidicoccales) have an MGG sequence at the N-terminus. Sequence alignments are available as Supplementary data.

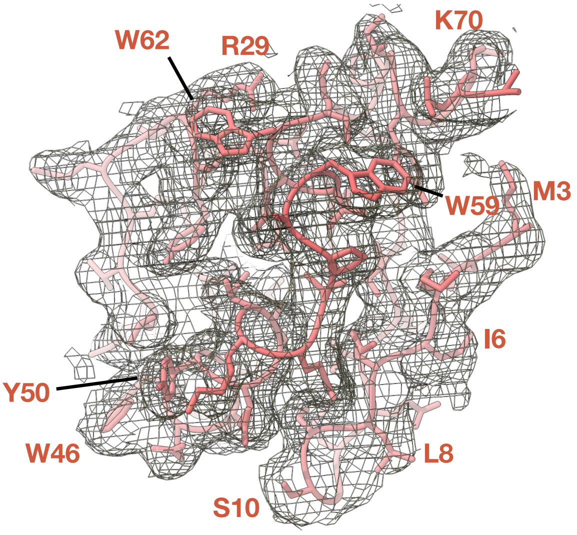


```
1        10         20         30         40         50         60         70
MEMLKIKLSSGREVEINDDTIAVLNEYVRTQMTLEELSKRLGLSGWEEAYELIKQVPAWVMWSPLPIYKKLA
```

Supplementary Figure 13: aS33

The view shows the quality of aS33 fitting into the cryo-EM map (multibody refined map EMDB-50445). aS33 constructed from the cryo-EM map is superimposable on an AlphaFold prediction with an rmsd of 0.58 Å for 57 C-alpha atoms compared.

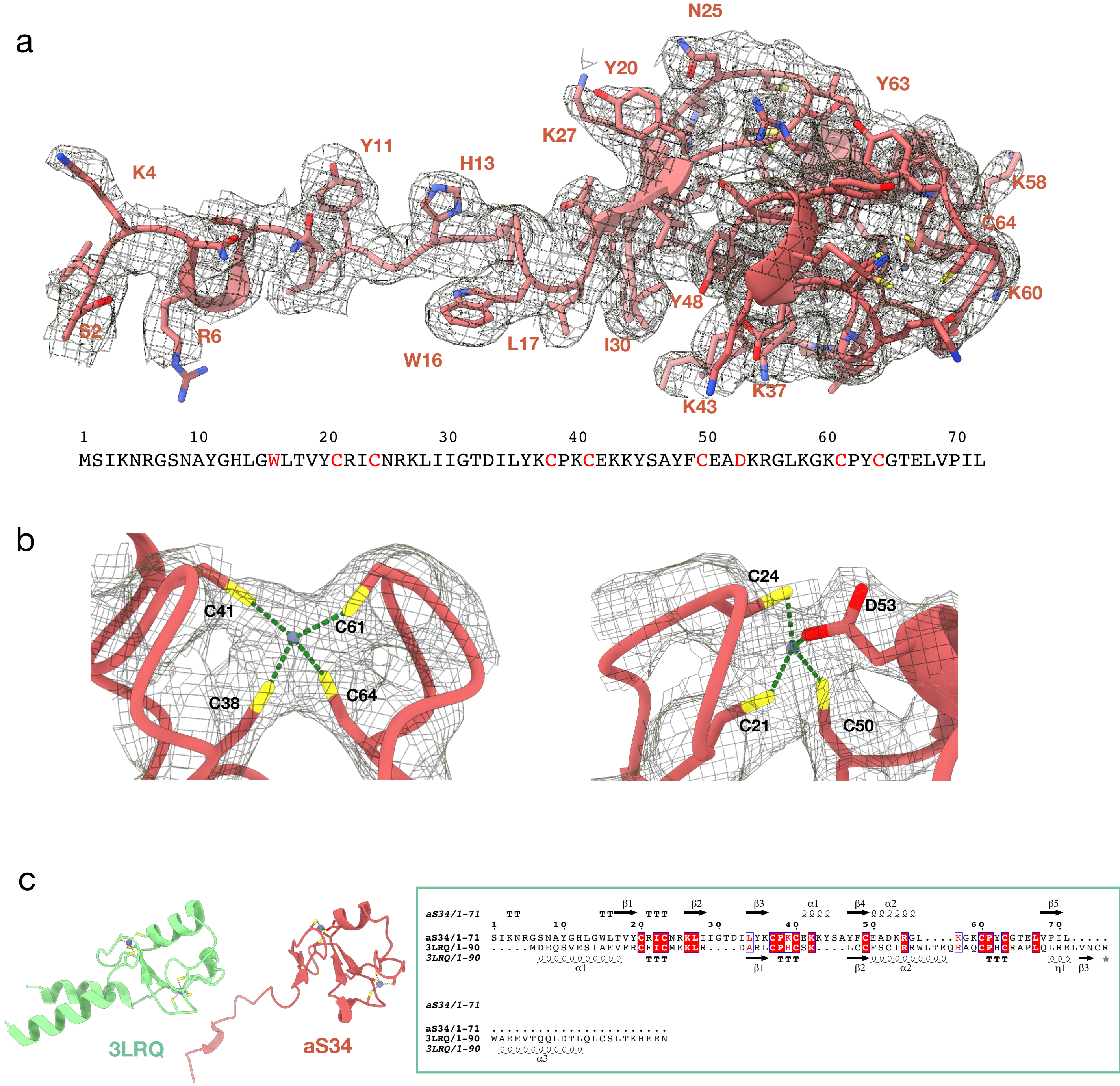

Supplementary Figure 14: aS34

a. The view shows the quality of aS34 fitting into the cryo-EM map (Local scale map EMDB 504445). aS34 constructed from the cryo-EM map is superimposable on an AlphaFold model with an rmsd of 0.53 Å for 54 C-alpha atoms compared.
b. Close-up views of the two zinc binding knuckles.
c. Superimposition of aS34 with human ubiquitin-protein ligase E3 RING domain (PDB 3LRQ). 3LRQ is in light green, aS34 in red. Rmsd 3LRQ to aS34, 2.9 Å for 217 atoms. Structural alignments were performed with Espript[39].

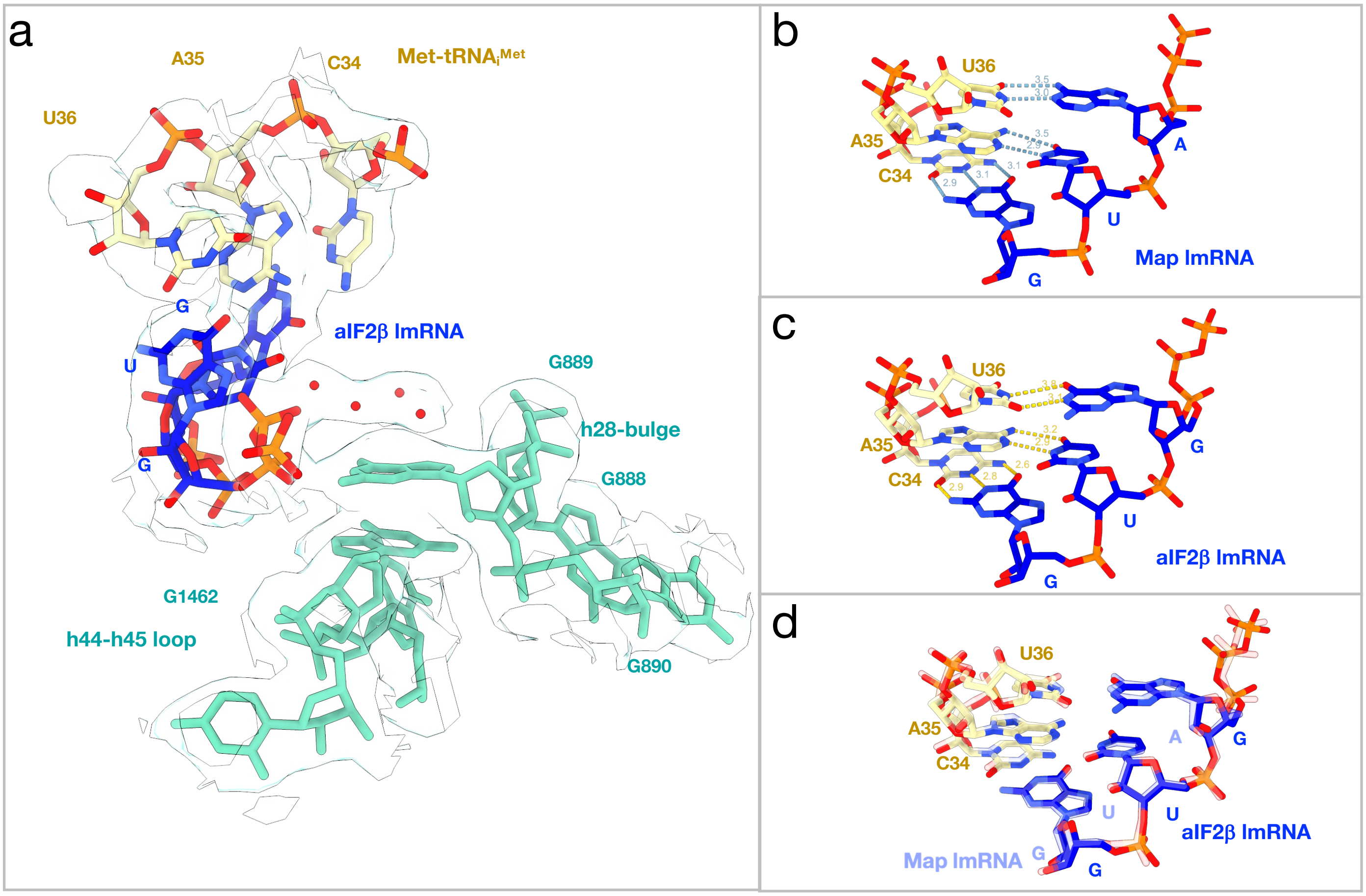

Supplementary Figure 15: P site interaction in DS3 and comparison with DS2

a. Close-up of the codon:anticodon interaction in IC-DS3. The cryo-EM map is represented to highlight the network of water molecules located above G889.
b. Close-up showing the AUG:CAU codon:anticodon interaction in IC-DS2.
c. Close-up showing the GUG:CAU codon:anticodon interaction in IC-DS3.
d. Superimposition of views B and C with view B shown in transparency. The view shows slight adjustment of the codon:anticodon interaction depending on the sequence of the start codon.

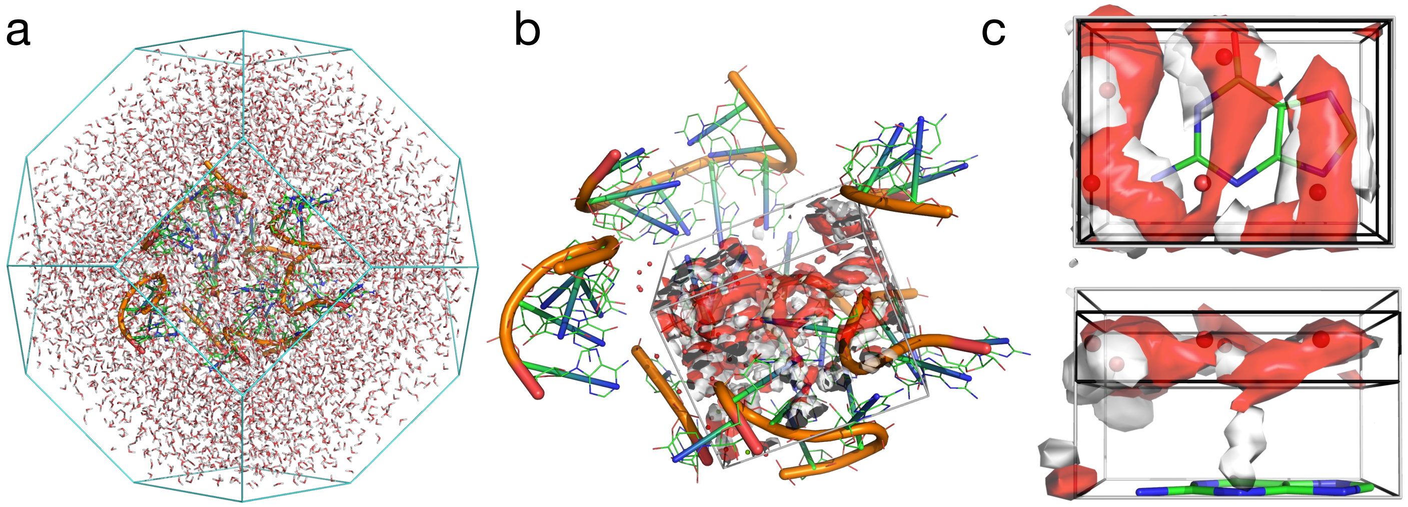

Supplementary Figure 16: Explicit water molecular dynamics simulation

a. Simulated system. The ribosomal molecular system was truncated at 12 Å around G889 nucleobase, immersed in a truncated octahedron box extending 10 Å away from the solute and filled with water molecules.
b. The view shows the simulated system along with water oxygen and hydrogen densities obtained by GIST.
c. Zoomed view on the region above G889 nucleobase. The view shows that the GIST predicted water oxygen (red) and hydrogen (white) densities fit with the experimentally placed water molecules (red spheres).

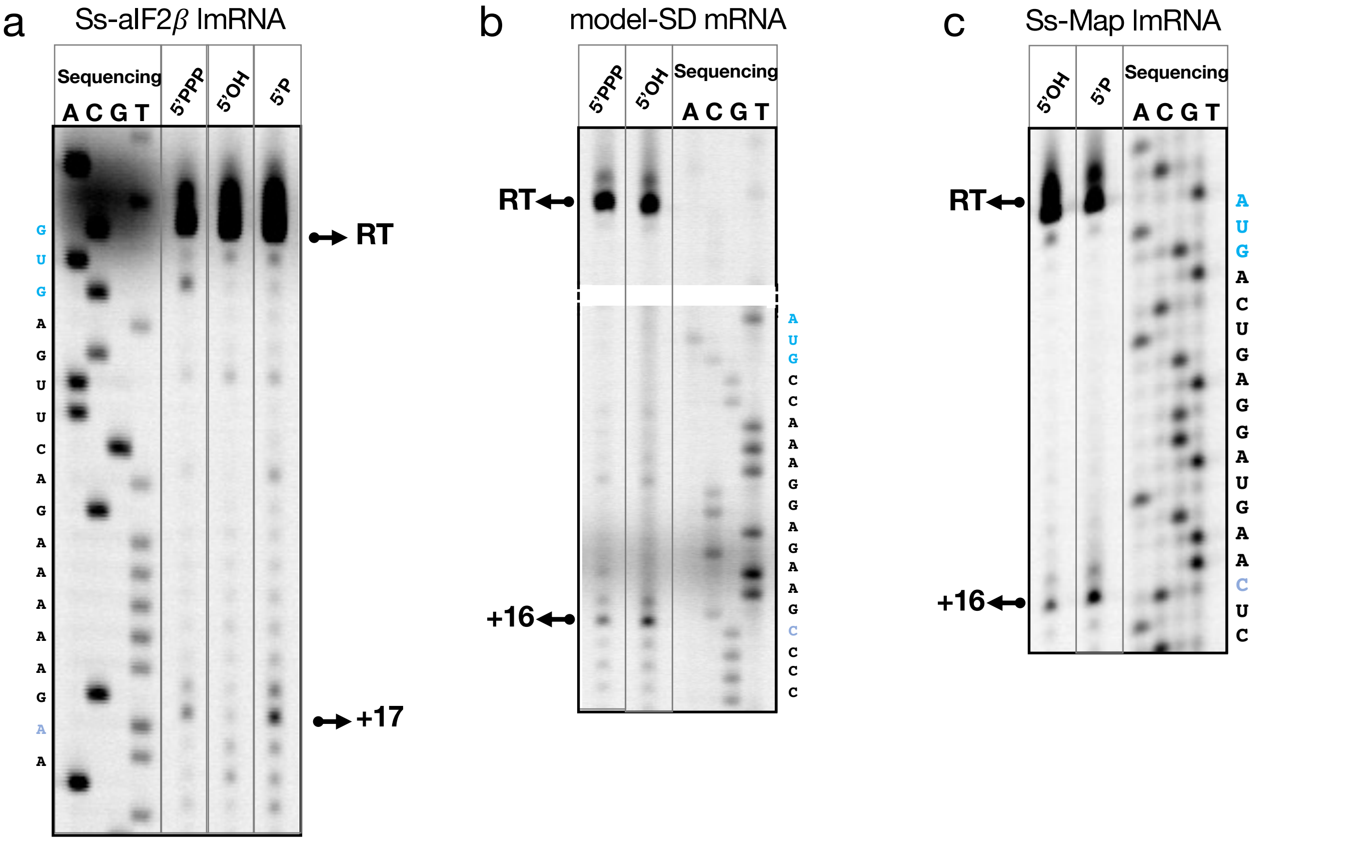

Supplementary Figure 17: Toeprinting analysis of 30S complexes assembled on mRNAs with a 5'PPP, 5'OH or 5'P end

a. Typical experiment using Ss-aIF2β lmRNA. Position of the initiation codon is indicated in blue. Position of the toeprinting signal observed at +17 is shown in light blue. The sequence of the mRNA complementary to the DNA sequence is shown on the left of the gel. Toeprinting signal obtained for a 30S:mRNA:Met-tRNA$_i^{Met}$:aIF2 complex where mRNA is 5'PPP, 5'OH, or 5'P, as indicated.
b. Same as panel a but using Model-SD mRNA.
c. Same as panel a but using Ss-MAP lmRNA. This lmRNA was produced by *in vitro* transcription using a Hammerhead ribozyme construct (see Methods). It therefore has a 5'-OH end. Lane 1 is with a 5'OH end and lane 2 is with a 5'P end added with polynucleotide kinase. Uncropped gels are shown in Supplementary data files.

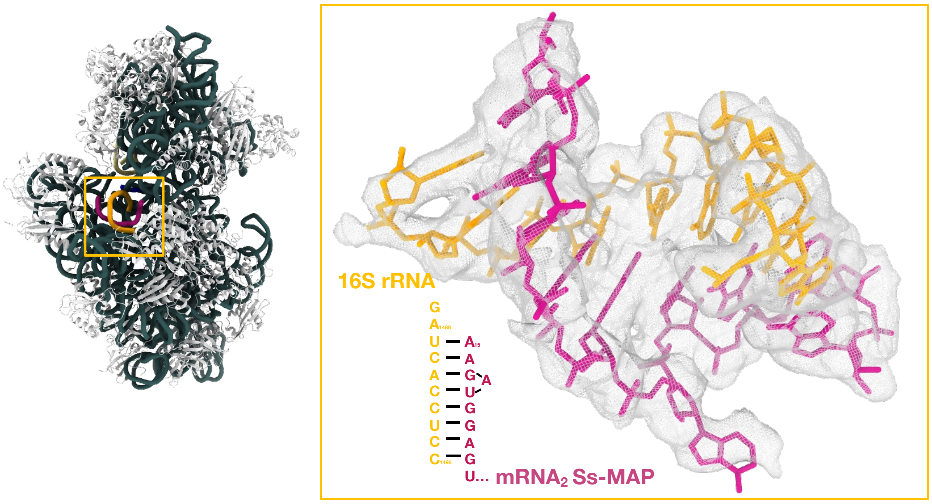

Supplementary Figure 18: mRNA exit channel in IC-DS2

The close up view shows the interaction of a second mRNA molecule (mRNA$_2$ Ss-MAP) with the anti SD sequence of the 16S rRNA.

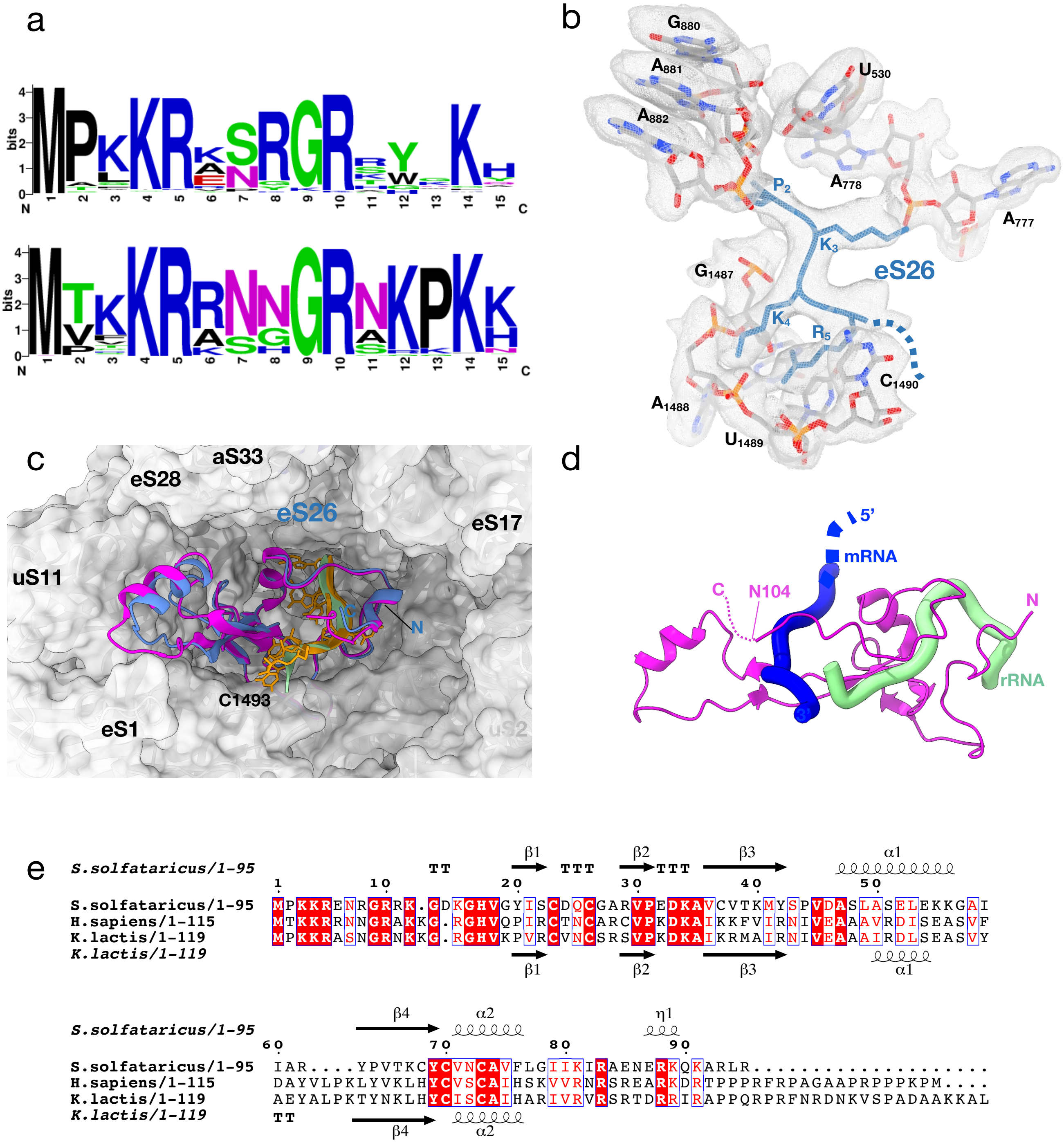

Supplementary Figure 19: archaeal and eukaryotic eS26

a. Archaeal and eukaryotic eS26 protein sequences were retrieved from Uniprot and aligned using Clustal Omega. The alignment was refined manually. 270 archaeal sequences were aligned. Average length of the protein is 95 amino acids. The sequence logo (Top) of the N-terminal residues shows a preference for the MPKKR sequence. The N-terminal peptide is tightly bound to the ribosome, as shown in view b. 1978 eukaryotic eS26 were aligned. 644 sequences containing unusual N-terminal regions were removed from the alignement. The logo shown at the bottom of the panel was calculated from 1334 remaining sequences. Average length of the protein is 115 amino acids. As compared to archaea, eukaryotic eS26 have longer C-terminal tail containing low complexity regions rich in proline residues. The C-tail (residues 97 to 104) of *K. lactis* eS26 contact the mRNA[32]. Sequence alignments are available as Supplementary data.

b. Binding of the Ss-eS26 N-terminal tail to 16S rRNA as observed in IC-DS3. The rRNA 3' end is shown in grey.

c. Structure of IC-DS3 with Ss-eS26 colored in blue. Human eS30 from 8G5Z[34] is colored in magenta. Ss-16S rRNA extremity is in orange and that of human 18S rRNA is in light green. The view shows a remarkable conservation between archaeal and eukaryotic eS26 structures as well as in the binding to the rRNA

d. Binding of *K. lactis* eS26 to rRNA and mRNA (PDB 6FYX)[32]. The view shows that the C-terminal tail of eS26 contacts the mRNA.

e. Structural alignment of *S. solfataricus* eS26 with the human and *K. lactis* proteins.

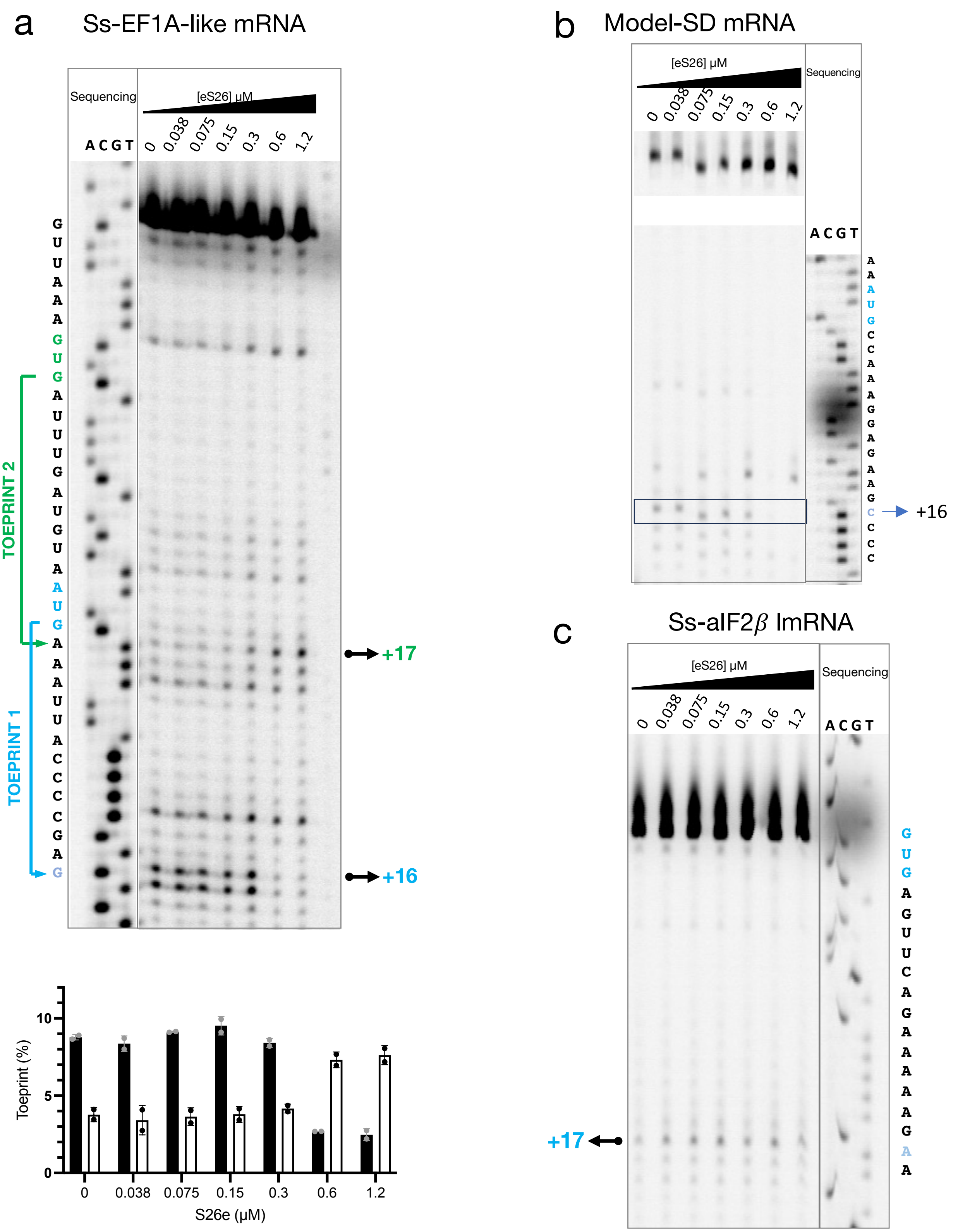

Supplementary Figure 20: high concentrations of eS26 destabilize the initiation complexes formed on leadered mRNAs

a. Influence of Ss-eS26 concentration on toeprinting signal intensity obtained with a 30S:mRNA:aIF2:Met-tRNA$_i^{Met}$ complex. The mRNA is Ss-EF1A-like. The two toeprinting positions discussed in the text are indicated. At high Ss-eS26 concentrations, the signal corresponding to toeprint 2 increases. The gel image shows a representative experiment. Below the gel image are the quantifications of the main bands (shown by arrows) corresponding to toeprint 1 (leadered start, full bars) or toeprint 2 (leaderless start, empty bars). Data are presented as means ± SD from two independent experiments. Dots show individual data points.
b. Same as view a but for Model-SD mRNA (representative experiment, see Figure 1 c)).
c. Same as view a but for leaderless Ss-aIF2β lmRNA (representative experiment, see Figure 1c). Source data are provided as a Source Data file. Uncropped gels are shown in Supplementary data files.

a

b

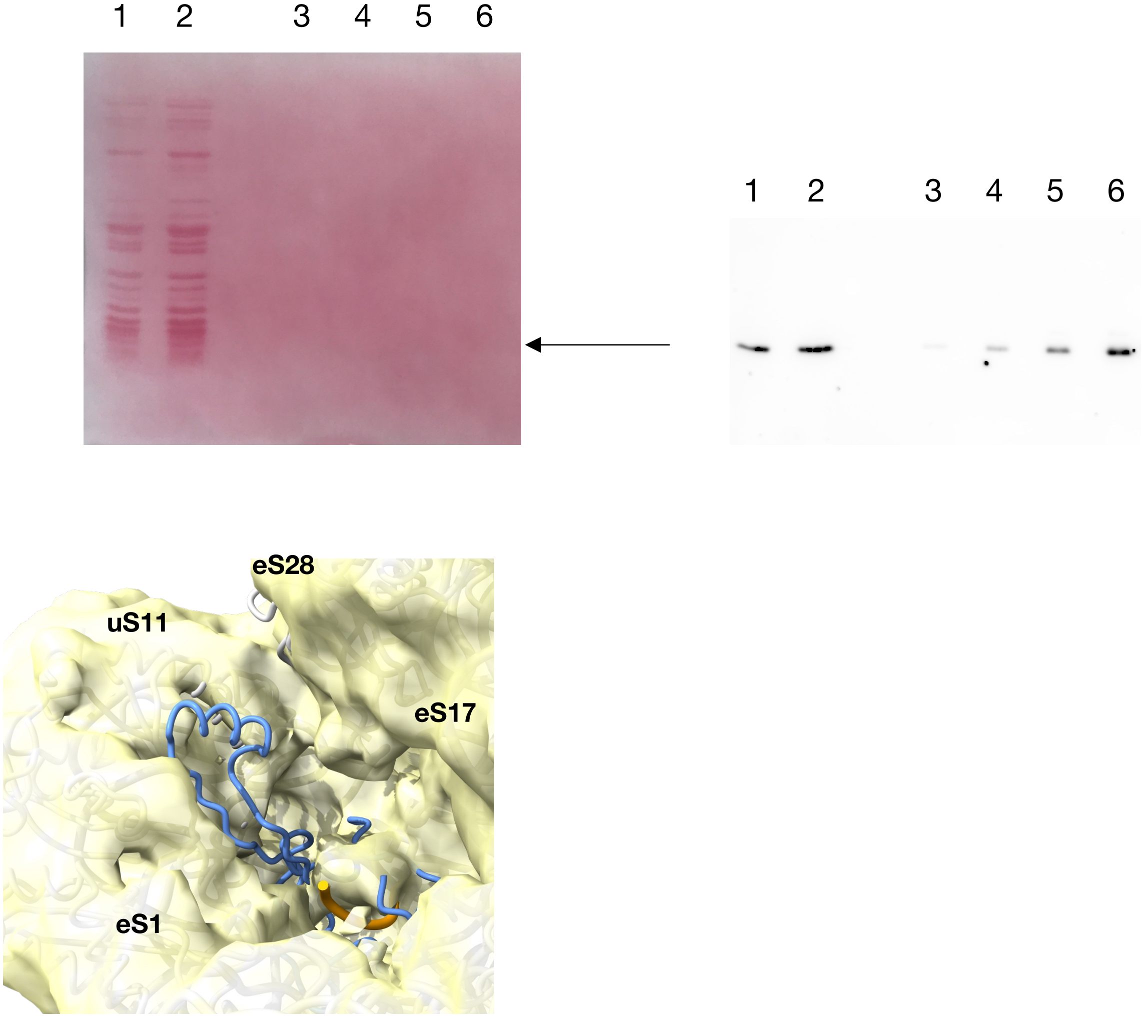

Supplementary Figure 21: eS26 is present in a minor fraction of purified Ss-30S subunits

a. Western blot analysis of purified Ss-30S subunits. Ss-30S were analyzed by SDS-PAGE, together with various amounts of purified Ss-eS26. For Western blot analysis, we used rabbit anti-Ss-eS26 antibodies revealed by peroxidase-coupled secondary antibodies (Sigma) and the Lumi-Light plus ECL system (Roche; right). Lanes were loaded with purified Ss-30S subunits (lane 1, 5 pmoles; lane 2, 10 pmoles) or purified Ss-eS26 (lane 3, 0.0625 pmoles (0.67 ng); lane 4, 0.125 pmoles; lane 5, 0.25 pmoles; lane 6, 0.5 pmoles). The experiment shows that *ca* 5 % of purified Ss-30S subunits contain eS26. Ponceau red staining of the membrane is shown on the left.

b. Cryo-EM analysis of purified Ss-30S. Purified Ss-30S subunits (3.4 µM) were activated during 30 min at 65°C, diluted to 100 nM and spotted onto a cryo-EM grid (Quantifoil R2.1 with 2 nm continuous carbon). Images were collected on an in-site Titan Themis cryo-microscope. Images were processed in Cryosparc (4158 good-quality images). As expected for empty 30S subunits, the head was highly mobile with respect to the body. Thus, particle were aligned on the body. This led to a cryo-EM map at 4.55 Å resolution for 408,560 particles. The DS3 structure (30S:aIF2b-lmRNA:tRNA) was fitted in the map. As shown in view b, eS26 (blue) can not be detected in the exit chamber even at low threshold values of the map. We then performed extensive trials of 3D classifications, not-focused or focused on various regions encompassing the eS26 site. This did not allow us to identify an eS26-containing class. This is consistent with the low amount of eS26 deduced from the Western blot analysis (panel a). Moreover, because eS26 is in equilibrium with the 30S, the necessary dilution to 100 nM before spotting on grids likely contributed, by mass action, to diminish even more the fraction of bound eS26.

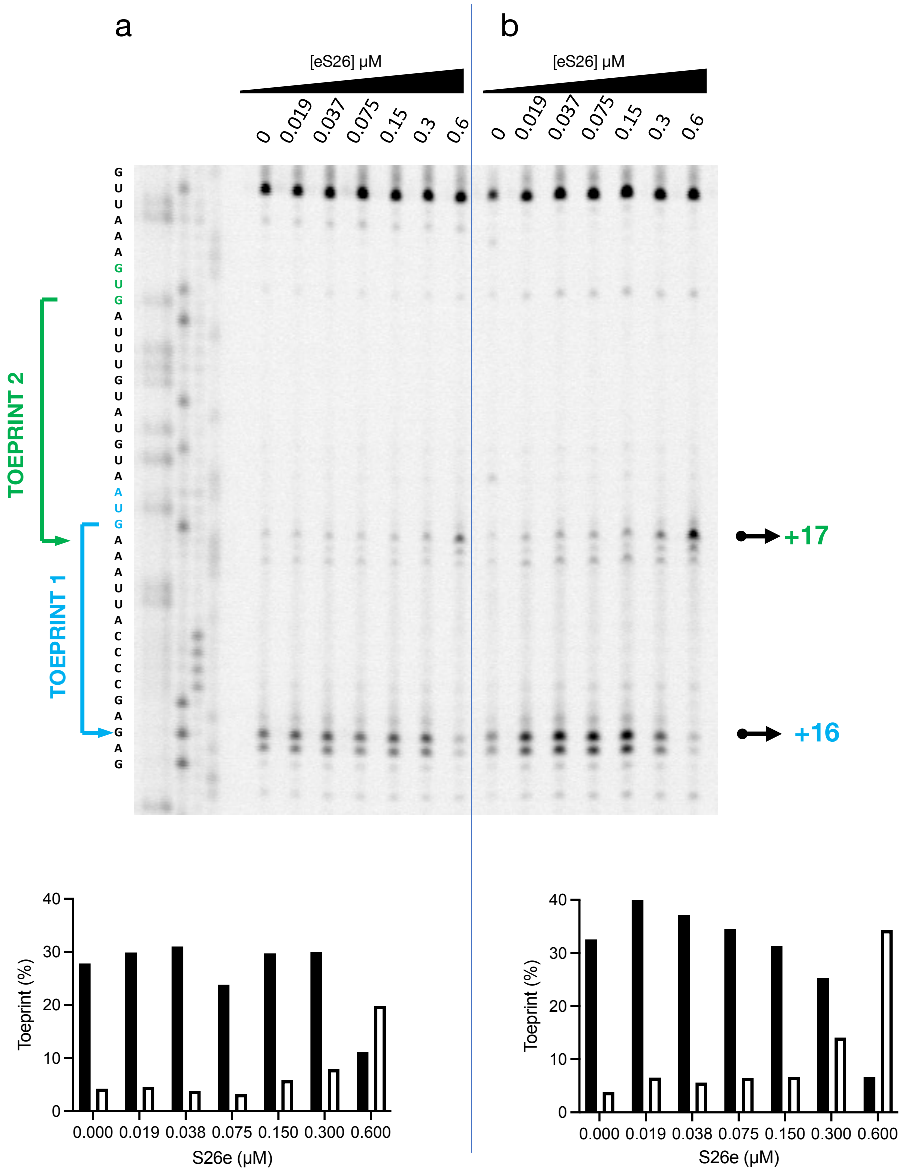

Supplementary Figure 22: preincubation of 30S with eS26 or adding eS26 as a factor have similar effects on toeprinting with Ss-EF1A-like mRNA.

a. Same as in Supplementary Figure 20a, except that final concentrations of aIF2 and Met-tRNA were 5μM.
b. Same as panel a but with 30S preincubated with eS26 5 minutes at 65°C before the toeprinting experiment (n=1). Indicated eS26 concentrations are those during reverse transcription. Below the gel image are the quantifications of the main bands (shown by arrows) corresponding to toeprint 1 (leadered start, full bars) or toeprint 2 (leaderless start, empty bars). Source data are provided as a Source Data file. Uncropped gels are shown in Supplementary data files.

SUPPLEMENTARY REFERENCES

1. Lazaridis T. Inhomogeneous Fluid Approach to Solvation Thermodynamics. 1. Theory. *The Journal of Physical Chemistry B* **102**, 3531-3541 (1998).
2. Nguyen CN, Young TK, Gilson MK. Grid inhomogeneous solvation theory: hydration structure and thermodynamics of the miniature receptor cucurbit[7]uril. *J Chem Phys* **137**, 044101 (2012).
3. Word JM, Lovell SC, Richardson JS, Richardson DC. Asparagine and glutamine: using hydrogen atom contacts in the choice of side-chain amide orientation. *J Mol Biol* **285**, 1735-1747 (1999).
4. Ryckaert J-P, Ciccotti G, Berendsen HJC. Numerical integration of the cartesian equations of motion of a system with constraints: molecular dynamics of n-alkanes. *Journal of Computational Physics* **23**, 327-341 (1977).
5. Darden T, York D, Pedersen L. Particle mesh Ewald: An N·log(N) method for Ewald sums in large systems. *The Journal of Chemical Physics* **98**, 10089-10092 (1993).
6. Loncharich RJ, Brooks BR, Pastor RW. Langevin dynamics of peptides: the frictional dependence of isomerization rates of N-acetylalanyl-N'-methylamide. *Biopolymers* **32**, 523-535 (1992).
7. Berendsen HJC, Postma JPM, van Gunsteren WF, DiNola A, Haak JR. Molecular dynamics with coupling to an external bath. *The Journal of Chemical Physics* **81**, 3684-3690 (1984).
8. Case DA*, et al.* AMBER 2021, University of California, San Francisco (2021).
9. Salomon-Ferrer R, Götz AW, Poole D, Le Grand S, Walker RC. Routine Microsecond Molecular Dynamics Simulations with AMBER on GPUs. 2. Explicit Solvent Particle Mesh Ewald. *Journal of Chemical Theory and Computation* **9**, 3878-3888 (2013).
10. Grand SML, Götz AW, Walker RC. SPFP: Speed without compromise - A mixed precision model for GPU accelerated molecular dynamics simulations. *Comput Phys Commun* **184**, 374-380 (2013).
11. Pérez A*, et al.* Refinement of the AMBER Force Field for Nucleic Acids: Improving the Description of α/γ Conformers. *Biophysical Journal* **92**, 3817-3829 (2007).
12. Zgarbová M*, et al.* Refinement of the Cornell et al. Nucleic Acids Force Field Based on Reference Quantum Chemical Calculations of Glycosidic Torsion Profiles. *Journal of Chemical Theory and Computation* **7**, 2886-2902 (2011).
13. Aduri R, Psciuk BT, Saro P, Taniga H, Schlegel HB, SantaLucia J. AMBER Force Field Parameters for the Naturally Occurring Modified Nucleosides in RNA. *J Chem Theory Comput* **3**, 1464-1475 (2007).
14. Meagher KL, Redman LT, Carlson HA. Development of polyphosphate parameters for use with the AMBER force field. *J Comput Chem* **24**, 1016-1025 (2003).
15. Jorgensen WL, Chandrasekhar J, Madura JD, Impey RW, Klein ML. Comparison of simple potential functions for simulating liquid water. *The Journal of Chemical Physics* **79**, 926-935 (1983).
16. Li P, Roberts BP, Chakravorty DK, Merz KM, Jr. Rational Design of Particle Mesh Ewald Compatible Lennard-Jones Parameters for +2 Metal Cations in Explicit Solvent. *Journal of Chemical Theory and Computation* **9**, 2733-2748 (2013).
17. Roe DR, Cheatham TE, III. PTRAJ and CPPTRAJ: Software for Processing and Analysis of Molecular Dynamics Trajectory Data. *Journal of Chemical Theory and Computation* **9**, 3084-3095 (2013).
18. Kalra K, Gorle S, Cavallo L, Oliva R, Chawla M. Occurrence and stability of lone pair-π and OH-π interactions between water and nucleobases in functional RNAs. *Nucleic Acids Res* **48**, 5825-5838 (2020).
19. Monestier A, Lazennec-Schurdevin C, Coureux PD, Mechulam Y, Schmitt E. Role of aIF1 in Pyrococcus abyssi translation initiation. *Nucleic Acids Res* **46**, 11061-11074 (2018).
20. Woese CR, Gupta R, Hahn CM, Zillig W, Tu J. The phylogenetic relationships of three sulfur dependent archaebacteria. *Syst Appl Microbiol* **5**, 97-105 (1984).
21. Kazan R*, et al.* Role of aIF5B in archaeal translation initiation. *Nucleic Acids Res* **50**, 6532-6548 (2022).
22. Sas-Chen A*, et al.* Dynamic RNA acetylation revealed by quantitative cross-evolutionary mapping. *Nature* **583**, 638-643 (2020).

23. Holvec S*, et al.* The structure of the human 80S ribosome at 1.9 Å resolution reveals the molecular role of chemical modifications and ions in RNA. *Nat Struct Mol Biol*, (2024).
24. Londei P, Teixido J, Acca M, Cammarano P, Amils R. Total reconstitution of active large ribosomal subunits of the thermoacidophilic archaebacterium Sulfolobus solfataricus. *Nucleic Acids Research* **14**, 2269-2285 (1986).
25. Cammarano P*, et al.* Archaebacterial elongation factor Tu insensitive to pulvomycin and kirromycin. *FEBS Lett* **148**, 255-259 (1982).
26. Zivanov J*, et al.* New tools for automated high-resolution cryo-EM structure determination in RELION-3. *eLife* **7**, e42166 (2018).
27. Barchet C, Fréchin L, Holvec S, Hazemann I, von Loeffelholz O, Klaholz BP. Focused classifications and refinements in high-resolution single particle cryo-EM analysis. *J Struct Biol* **215**, 108015 (2023).
28. Van Heel M., Keegstra W., G. SW, J. VBEF. Arthropod hemocyanin studied by image analysis. *Life Chem Rep Suppl* 69-73 (1982).
29. Saxton WO, Baumeister W. The correlation averaging of a regularly arranged bacterial cell envelope protein. *J Microsc* **127**, 127-138 (1982).
30. Rosenthal PB, Henderson R. Optimal determination of particle orientation, absolute hand, and contrast loss in single-particle electron cryomicroscopy. *J Mol Biol* **333**, 721-745 (2003).
31. Punjani A, Rubinstein JL, Fleet DJ, Brubaker MA. cryoSPARC: algorithms for rapid unsupervised cryo-EM structure determination. *Nature Methods* **14**, 290-296 (2017).
32. Llacer JL*, et al.* Translational initiation factor eIF5 replaces eIF1 on the 40S ribosomal subunit to promote start-codon recognition. *Elife* **7**, e39273 (2018).
33. Thomas JM*, et al.* A Chemical Signature for Cytidine Acetylation in RNA. *J Am Chem Soc* **140**, 12667-12670 (2018).
34. Holm M*, et al.* mRNA decoding in human is kinetically and structurally distinct from bacteria. *Nature* **617**, 200-207 (2023).
35. Waterhouse A, Procter J, Martin D, Clamp M, Barton G. Jalview Version 2-a multiple sequence alignment editor and analysis workbench. *Bioinformatics* **25**, 1189-1191 (2009).
36. Martín-Villanueva S, Gutiérrez G, Kressler D, de la Cruz J. Ubiquitin and Ubiquitin-Like Proteins and Domains in Ribosome Production and Function: Chance or Necessity? *International Journal of Molecular Sciences* **22**, 4359 (2021).
37. Baker RT, Williamson NA, Wettenhall REH. The Yeast Homolog of Mammalian Ribosomal Protein S30 Is Expressed from a Duplicated Gene without a Ubiquitin-like Protein Fusion Sequence: EVOLUTIONARY IMPLICATIONS*. *Journal of Biological Chemistry* **271**, 13549-13555 (1996).
38. van den Heuvel J*, et al.* Processing of the ribosomal ubiquitin-like fusion protein FUBI-eS30/FAU is required for 40S maturation and depends on USP36. *Elife* **10**, (2021).
39. Gouet P, Courcelle E, Stuart DI, Metoz F. ESPript: analysis of multiple sequence alignments in PostScript. *Bioinformatics* **15**, 305-308 (1999).
