# Supplementary material for: Structures of Saccharolobus solfataricus initiation complexes with leaderless mRNAs highlight archaeal features and eukaryotic proximity
Source: Nat Commun. 2025 Jan 2;16:348. doi: 10.1038/s41467-024-55718-5 (PMC11698992; doi:10.1038/s41467-024-55718-5)
Supplement: Supplementary file 2 — Description of Additional Supplementary Files [file 41467_2024_55718_MOESM2_ESM.pdf]

Description of Additional Supplementary Files

Title: Supplementary Data 1
Description: The Supplementary Data 1 contains five folders and one file. One folder is for molecular dynamics simulation. This folder contains PDB file of initial coordinates, PDB file of initial coordinates with water box, AMBER prep file for A3P nucleotide, AMBER input files for minimization and MD, PDB file of final coordinates with water box, DX files of GIST water oxygen and hydrogen densities, Pymol file for visualization and a Readme file. Four folders are for sequence alignments files. Finally, the supplementary file contains uncropped gels related to supplementary figures.
