# Supplementary material for: Structures of Saccharolobus solfataricus initiation complexes with leaderless mRNAs highlight archaeal features and eukaryotic proximity
Source: Nat Commun. 2025 Jan 2;16:348. doi: 10.1038/s41467-024-55718-5 (PMC11698992; doi:10.1038/s41467-024-55718-5)
Supplement: Supplementary file 3 — Supplementary Data 1 [file 41467_2024_55718_MOESM3_ESM.zip › Supplementary Data/uncropped gels Supplementary Figures.pdf]

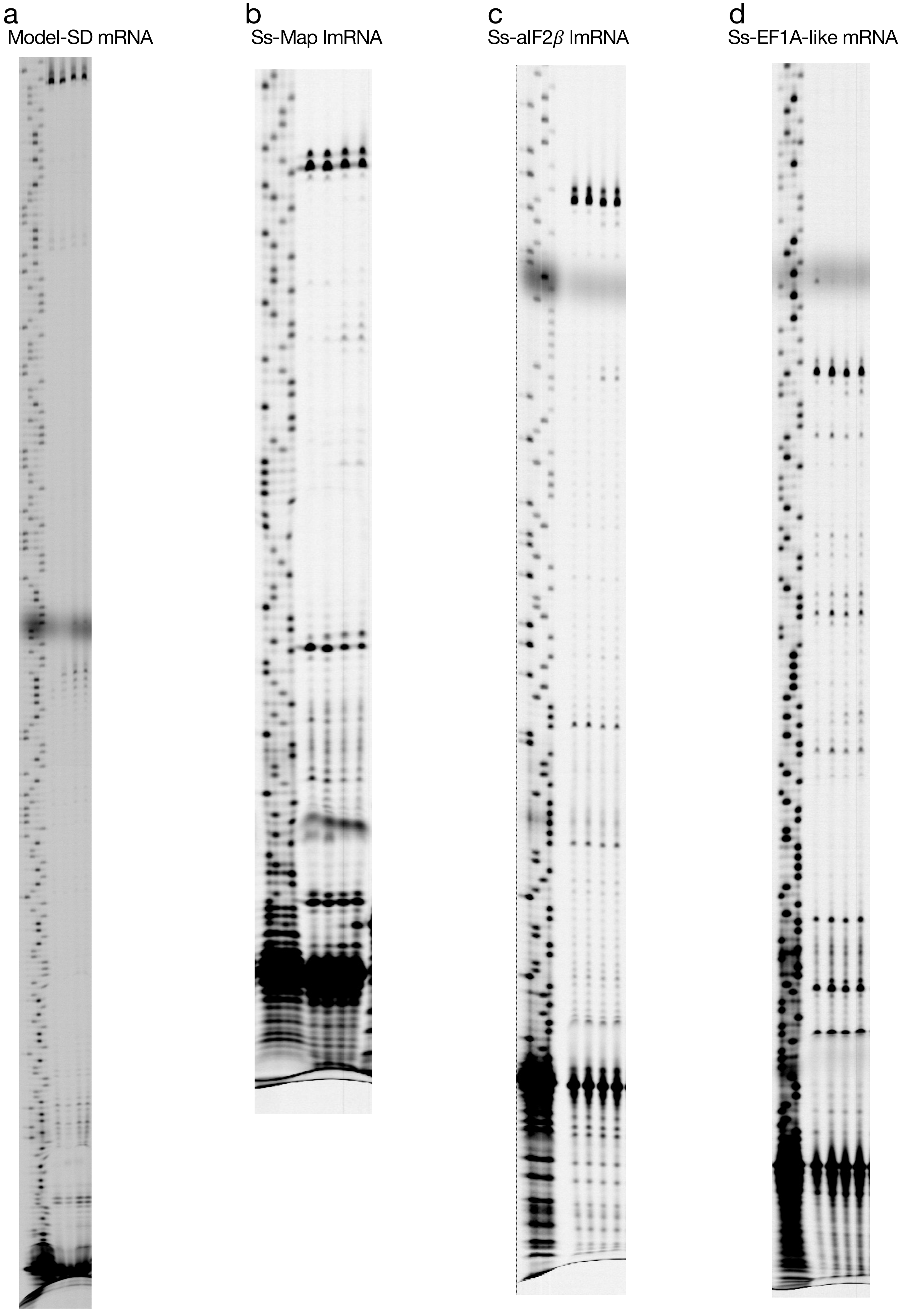

Uncropped gels for Supplementary Figure 3

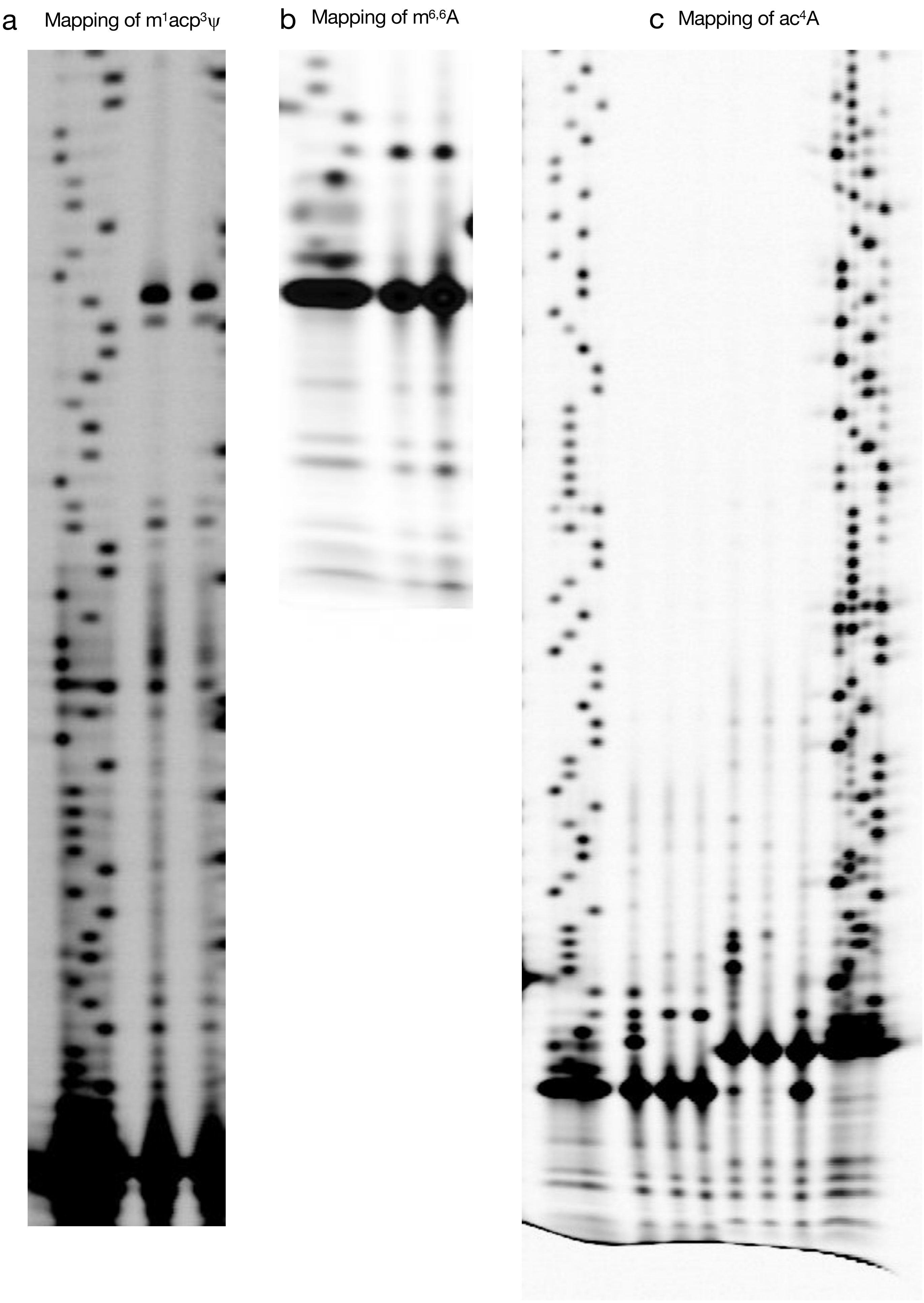

Uncropped gels for Supplementary Figure 10

a Ss-aIF2β lmRNA

b model-SD mRNA

c Ss-Map lmRNA

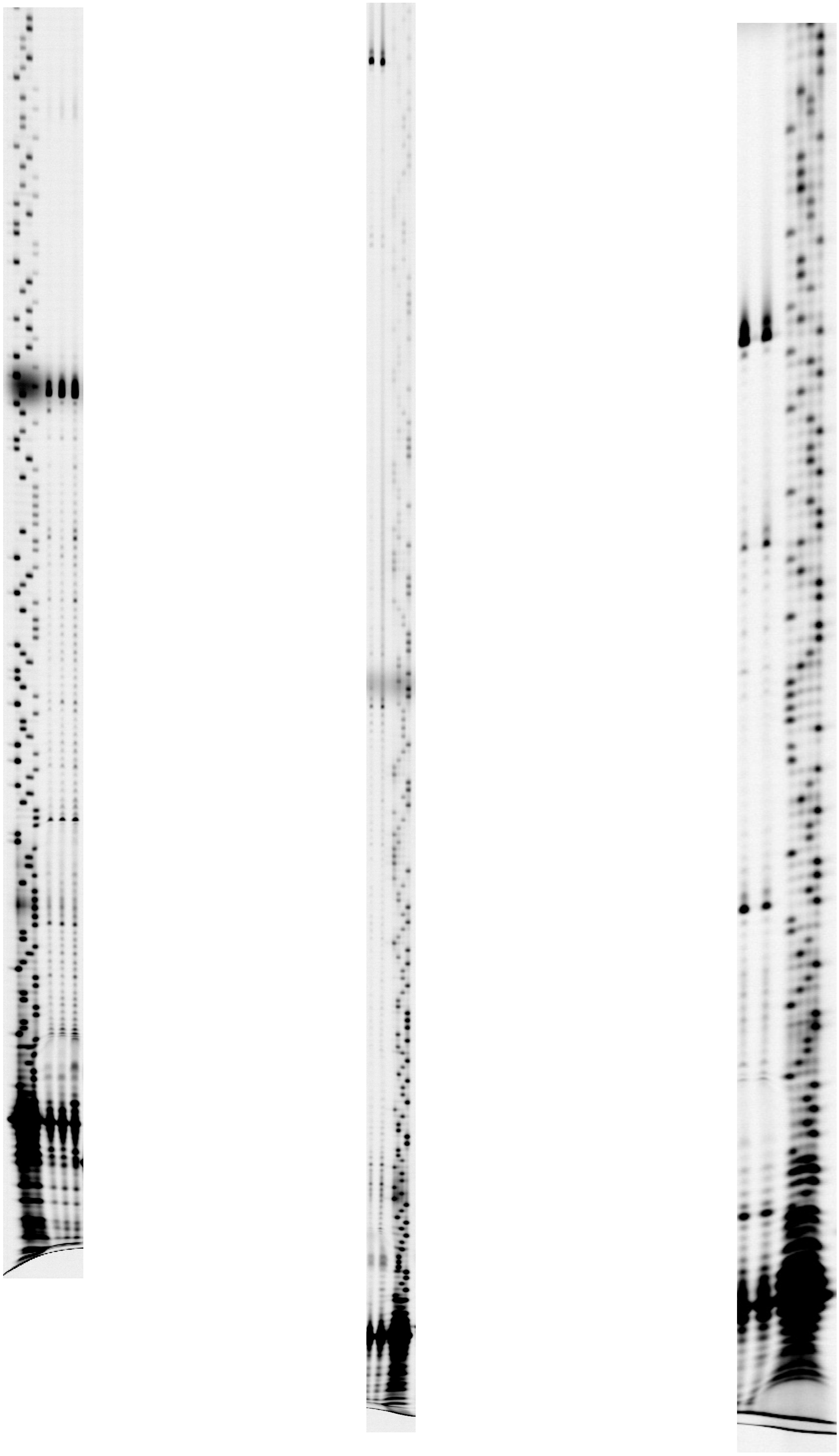

Uncropped gels for Supplementary Figure 17

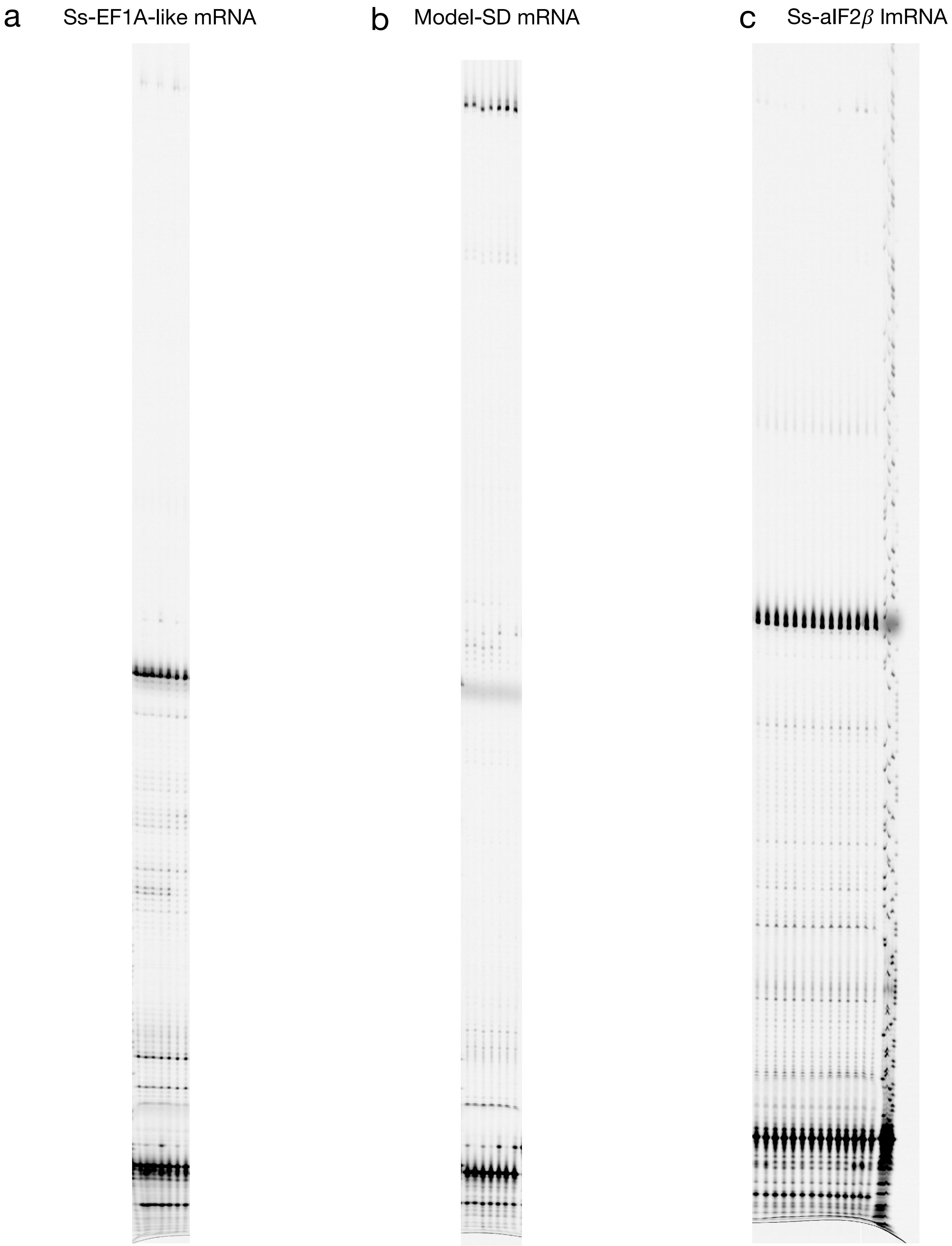

Uncropped gels for Supplementary Figure 20

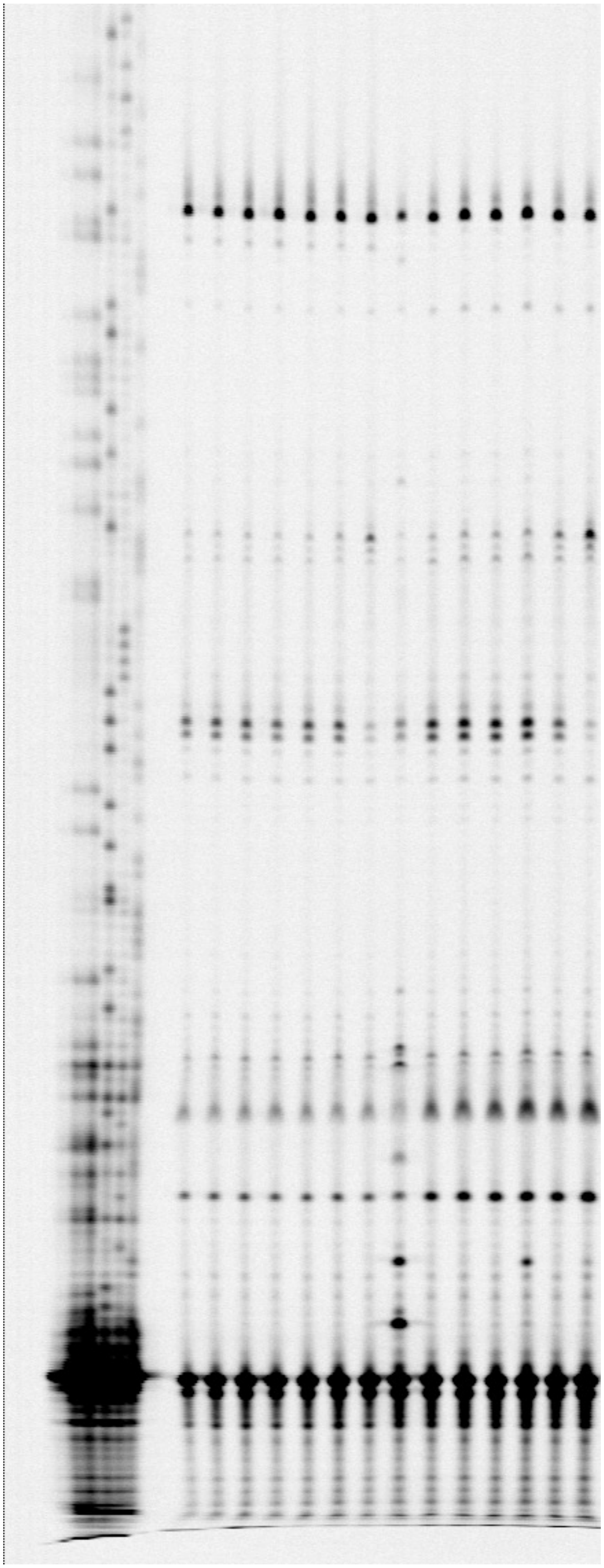

Uncropped gels for Supplementary Figure 22
